# Increased A1 astrocyte activation‐driven hippocampal neural network abnormality mediates delirium‐like behavior in aged mice undergoing cardiac surgery

**DOI:** 10.1111/acel.14074

**Published:** 2023-12-28

**Authors:** Wenxue Liu, Min Jia, Keyin Zhang, Jiang Chen, Xiyu Zhu, Ruisha Li, Zhenjun Xu, Yanyu Zang, Yapeng Wang, Jun Pan, Daqing Ma, Jianjun Yang, Dongjin Wang

**Affiliations:** ^1^ Department of Cardio‐Thoracic Surgery, Institute of Cardiothoracic Vascular Disease, Nanjing Drum Tower Hospital, Affiliated Hospital of Medical School Nanjing University Nanjing China; ^2^ Department of Anesthesiology, Pain and Perioperative Medicine The First Affiliated Hospital of Zhengzhou University Zhengzhou China; ^3^ Ministry of Education Key Laboratory of Model Animal for Disease Study, Model Animal Research Center, Department of Neurology, Drum Tower Hospital, Medical School Nanjing University Nanjing China; ^4^ Ministry of Education Key Laboratory of Model Animal for Disease Study, Model Animal Research Center Nanjing University Nanjing China; ^5^ Department of Cardio‐Thoracic Surgery, Nanjing Drum Tower Hospital Chinese Academy of Medical Sciences & Peking Union Medical College Nanjing China; ^6^ Division of Anaesthetics, Pain Medicine and Intensive Care, Department of Surgery and Cancer, Faculty of Medicine Imperial College London, Chelsea and Westminster Hospital London UK; ^7^ Perioperative and Systems Medicine Laboratory, Children’s Hospital, Zhejiang University School of Medicine National Clinical Research Center for Child Health Hangzhou China

**Keywords:** A1 astrocytes, cardiac surgery, delirium, neural networks

## Abstract

Delirium is the most common neurological complication after cardiac surgery with adverse impacts on surgical outcomes. Advanced age is an independent risk factor for delirium occurrence but its underlying mechanisms are not fully understood. Although increased A1 astrocytes and abnormal hippocampal networks are involved in neurodegenerative diseases, whether A1 astrocytes and hippocampal network changes are involved in the delirium‐like behavior of aged mice remains unknown. In the present study, a mice model of myocardial ischemia–reperfusion mimicking cardiac surgery and various assessments were used to investigate the different susceptibility of the occurrence of delirium‐like behavior between young and aged mice and the underlying mechanisms. The results showed that surgery significantly increased hippocampal A1 astrocyte activation in aged compared to young mice. The high neuroinflammatory state induced by surgery resulted in glutamate accumulation in the extrasynaptic space, which subsequently decreased the excitability of pyramidal neurons and increased the PV interneurons inhibition through enhancing N‐methyl‐D‐aspartate receptors' tonic currents in the hippocampus. These further induced the abnormal activities of the hippocampal neural networks and consequently contributed to delirium‐like behavior in aged mice. Notably, the intraperitoneal administration of exendin‐4, a glucagon‐like peptide‐1 receptor agonist, downregulated A1 astrocyte activation and alleviated delirium‐like behavior in aged mice, while IL‐1α, TNF‐α, and C1q in combination administered intracerebroventricularly upregulated A1 astrocyte activation and induced delirium‐like behavior in young mice. Therefore, our study suggested that cardiac surgery increased A1 astrocyte activation which subsequently impaired the hippocampal neural networks and triggered delirium development.

## Graphical abstract

Cardiac surgery increased microglia and A1 astrocyte activation and then led to the accumulation of glutamate, which ultimately impaired the hippocampal neural networks through enhancing NMDAR tonic activation, contributing to delirium‐like behavior in aged mice.

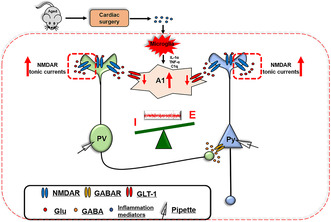

**Abbreviations**
ACSF: Artificial cerebrospinal fluid
AD: Alzheimer's disease
ALS: Amyotrophic Lateral Sclerosis
AMPAR: α‐amino‐3‐hydroxy‐5‐methyl‐4‐isoxazolepropionic acid receptor
AP: Anterior‐Posterior
Con: Control
DI: Discrimination index
DV: Dorsal‐Ventral
ELISA: Enzyme‐linked immunosorbent assay
EPM: Elevated plus maze test
GABA: Gamma‐aminobutyric acid
GLAST‐1: Glutamate arginine transporter
GLP‐1R: Glucagon‐like peptide‐1 receptor
GLT‐1: Glutamate transporter‐1
HPLC: High‐performance liquid chromatography
i.c.v.: Intracerebroventricular
IF: Immunofluorescent staining
IR: Ischemia‐reperfusion
LAD: left anterior descending artery
LFP: Local field potentials
mEPSCs: Miniature excitatory postsynaptic currents
mIPSC: Miniature inhibitory postsynaptic currents
ML: Medial‐Lateral
NMDAR: N‐Methyl‐D‐aspartic acid receptor
NS: normal saline
OA: open arm
OFT: Open field test
ORT: Object recognition test
PD: Parkinson's disease
POD: Postoperative delirium
PV: parvalbumin
RT: room temperature
WB: Western Blot
YMT: Y maze test

## 1 INTRODUCTION

Postoperative delirium (POD) is the most common neurological complication following cardiac surgery. It is characterized by acute, and fluctuating changes in consciousness, attention, and cognitive function with an incidence of 11–46% and was reported to be closely associated with increased postoperative mortality, longer hospital stay and costs, and developed long‐term cognitive dysfunction (Avidan et al., 2017; Inouye et al., 2014; Kunicki et al., 2023). Growing evidence suggests that advanced age is an independent risk factor for the incidence of delirium (Chaiwat et al., 2019; Kukreja et al., 2015). However, due to the limited study, the mechanisms of POD in aged patients following surgery including cardiac surgery remain elusive.

Previous preclinical and clinical studies demonstrated that systemic inflammatory responses following anesthesia and surgery resulted in neuroinflammation and ultimately damaged brain function (Alam et al., 2018; Jin et al., 2020; Kazmierski et al., 2014; Vizcaychipi et al., 2014; Zhu et al., 2021). Microglia and astrocytes play a crucial role in modulating neuroinflammation (Goshi et al., 2020; Jha et al., 2019). It has been demonstrated that the activation of microglia led to the secretion of inflammatory mediators such as IL‐1α, TNF‐α, and C1q. These worked in conjunction to activate the A1 phenotype of astrocytes which were neurotoxic and adversely affected the functions of neurons within the central nervous system (Liddelow et al., 2017). In addition, the basal activated level of A1 astrocytes is high and they were easily upregulated under stimulation in normal aging mice (Clarke et al., 2018). Furthermore, a high level of A1 astrocyte activation was found in neurodegenerative diseases, such as Alzheimer's disease (AD), Parkinson's disease (PD), depression, and amyotrophic lateral sclerosis (ALS) (Li et al., 2019; Liddelow et al., 2017; Yun et al., 2018; Zhang et al., 2020). Considering that aging, neuronal injury, and inflammatory responses are all considered to be risk factors for delirium development (Inouye et al., 2014). It is noteworthy that cardiac surgery can intensify systematic inflammatory responses. This was primarily due to the extended operative time, blood contact with the expansive foreign surface of the cardiopulmonary bypass circuit, and the ischemia–reperfusion injury (Laffey et al., 2002; Paparella et al., 2002). Therefore, A1 astrocyte activation and consequences may very likely contribute to the development of delirium after cardiac surgery.

Glutamate is the main excitatory neurotransmitter in the central nervous system, and its highly abnormal action is closely entwined with the occurrence of AD, depression, and PD (Hynd et al., 2004; Iovino et al., 2020; Rubio‐Casillas & Fernandez‐Guasti, 2016). Normally, astrocytes are responsible for the uptake of 90% glutamate from extrasynaptic space through glutamate transporter 1 (GLT‐1) and glutamate arginine transporter (GLAST‐1), and subsequently prevent neuronal damage from glutamate excitatory toxicity (John et al., 2012). Reduced uptake of glutamate by astrocytes led to the accumulation of glutamate in the extrasynaptic space, which caused neuronal over‐firing through high N‐methyl‐D‐aspartic acid receptor (NMDAR) activation (Hanson et al., 2019; Miller et al., 2014; Talantova et al., 2013). Previous studies demonstrated that the ability of A1 astrocytes to clear glutamate was compromised under disease conditions (Li et al., 2019), and brain electrical activity was abnormal in delirious patients (Hunter et al., 2020; Kimchi et al., 2019), suggesting that increased A1 astrocyte activation may impair the neural networks, and then contribute to the occurrence of delirium but warrants further study. Hence, this study was designed to investigate the impact of aging on delirium and underlying mechanisms after cardiac surgery in mice.

## 2 MATERIALS AND METHODS

### 2.1 Animals

Two months male C57BL/6 mice were purchased from Gempharmatech Inc (Nanjing, China), 20 months male C57BL/6 mice were purchased from Jiangsu Aniphe Biolaboratory Inc. (Nanjing, China), parvalbumin (PV)‐iCre mice and tdTomato mice were gifted by Prof. Yun Stone Shi (Medical School of Nanjing University). PV‐tdTomato mice were mutated by the crossing of the PV‐iCre mice and tdTomato mice. They were housed at 12 light/dark cycles with food and water ad libitum. For surgical procedures, mice were rapidly intubated following induction with 4% isoflurane and then maintained with 1.5–2% isoflurane. Their body temperature was kept around 37°C with a heating pad and their eyes were protected with chlortetracycline eye ointment throughout the procedure. All the animal experimental procedures in this study were approved by the Institutional Ethics Committee of Nanjing Drum Tower Hospital.

### 2.2 Myocardial ischemia–reperfusion (IR) as a surgery model

The myocardial IR experiment was performed as described previously with minor modifications (Curaj et al., 2015). Briefly, under anesthesia, their lungs were ventilated with a small animal ventilator (SAR‐830/AP; CWE Inc, PA, USA). A lateral thoracotomy was made between the fourth and fifth left rib and the left ventricle was then exposed gently. The ligation of the left anterior descending artery (LAD) was made immediately and it was removed 45 min later. The ischemia and reperfusion were confirmed under a stereomicroscope view of heart muscle color and contractility changes. After the procedure, chest wall muscle and skin were sutured separately.

### 2.3 Cannulas and microfilament array electrode implantation and intracerebroventricular injection

Under anesthesia, another cohort of mice was placed on a stereotaxic instrument (68025, RWD Life Science Co., Ltd, Shenzhen, China) for the cannula or 8‐channel microfilament array electrodes implementation (coordination: anterior‐posterior (AP) – 2.3 mm, medial‐lateral (ML) – 1.5 mm, and dorsal‐ventral (DV) – 1.5 mm) for microdialysis or electrophysiology recording from the CA1 region, or for intracerebroventricular (i.c.v.) injection (AP – 0.58 mm, ML − 1.25 mm, and DV – 2.25 mm) 7 days before myocardial IR. After the implantation, screws and glass ionomer cement were used to fix the cannula and microfilament array electrodes to the skull. According to the designated groups, 2 μL of artificial cerebrospinal fluid (ACSF) or 2 μL of TNF‐α (3 ng), IL‐1α (30 ng), and C1q (400 ng) in combination dissolved in ACSF was intracerebroventricularly injected with microsyringe at 1 μL per minute. The doses of inflammatory mediators were determined according to the previous studies (Liddelow et al., 2017; Palin et al., 2007) and/or our preliminary experiments.

### 2.4 Behavioral tests

#### 2.4.1 Open‐field test (OFT)

The mice were placed in the center of a 40 × 40 × 40 cm open‐field chamber and allowed to move freely for 5 min. The total distance, moving speed, and time spent in the center were calculated.

#### 2.4.2 Elevated plus maze (EPM) test

The EPM consists of two open arms and two closed arms, shaped in the cross with a 5 × 5 cm center region and elevated 50 cm from the floor. The dimension of each arm is 35 × 5 cm^2^, and closed arms were enclosed with 15 cm height walls. The mice were gently placed in the center region by facing the open arm. The total distance and the percentage of time spent in the open arms were analyzed.

#### 2.4.3 Y maze test (YMT)

The apparatus consists of three identical arms, length × width × height is 30 × 10 × 15 cm, and the angle between adjacent arms is 120°. Three arms were defined as A, B, and C, respectively. They were gently placed in the central region and allowed to move freely for 5 min. The number and order of entry into each arm were recorded and analyzed, and then calculated the alteration score. Alteration score = alteration/(total number of arms entries–2). The alteration was defined as entering arms in successions, such as ABC, ACB, BAC, or CAB.

#### 2.4.4 Object recognition test (ORT)

The ORT apparatus is an open field apparatus, length × width × height is 40 × 40 × 45 cm. Two objects with different shapes and colors blocks were used to be identified. Mice were gently held for more than 1 min and placed in the apparatus for more than 5 min twice every day at 8‐h intervals to avoid stress. The procedures were conducted 3 consecutive days before the formal experiment. The mice were placed in the experiment room to familiarize themselves with the environment 24 h before starting the experiment. The experiment was divided into training and testing sessions. For the training session, mice were presented with two identical blocks A and B and were allowed to explore freely for 10 min. The test session was carried out 24 h later, block A was replaced with block C, a new object with different colors and shapes compared with A and B, and the mice were allowed to explore freely for 10 min. Mice are color blind and need to be considered during the color selection. The exploration time of each block and discrimination index (DI) was analyzed. DI = exploration time of C/(exploration time of B + exploration time of C).

For all the above tests, their movements were tracked with Supermaze software (XR‐Xmaze, Shanghai Xinruan Information Technology Co., LTD, Shanghai, China). And the apparatus was cleaned with 30% ethanol to avoid the influence of residual odor between each trial.

### 2.5 Immunofluorescent staining (IF)

Mice were anesthetized with 5% isoflurane and transcardially perfused with cold PBS followed by isometric 4% PFA, and then the brains were harvested and postfixed in 4% PFA overnight at 4°C. The brains were dehydrated with 30% sucrose twice, and then embedded in OCT and frozen at −80°C. Slices (30 μm) were made using a freezing microtome. The slices were rinsed 3 × 5 min with PBS and blocked with 5–10% goat serum or donkey serum containing 0.3% Triton X‐100 for 1 h at room temperature (RT), and then incubated with primary antibodies including GFAP (1:500, ab68428; Abcam), C3d (15 μg/mL, AF2655‐SP; R&D), CD68 (1:500, 137,001; BioLegend), IBA1 (1:500, ab178846; Abcam), PV (1:500, MAB1572; Sigma Aldrich), and GAD65/67 (1:500, Ab183999; Abcam) overnight at 4°C. The slices were rinsed 3 × 5 min with PBS, and then incubated with Goat Anti‐Rabbit Alexa Fluor 488 (1:1000, Ab150077; Abcam), Goat Anti‐Mouse Alexa Fluor 488 (1:1000, Ab150113; Abcam), Goat Anti‐Rat Alexa Fluor 594 (1:1000, Ab150160; Abcam), or Donkey Anti‐Goat Alexa Fluor 647 (1:1000, Ab150131; Abcam) secondary antibody according to primary antibody at RT for 1 h. The images were captured by Olympus confocal microscope FV3000 with *z*‐stack using 40× oil objective. For each slice, 3–5 astrocytes or 3–5 microglia from CA1 stratum radiatum were randomly selected, and the GFAP, C3d, and DAPI or IBA1, CD68, and DAPI were 3D‐reconstructed using the surface rendering function in Imaris 10.0.0. The C3d inside GFAP and CD68 inside IBA1 were filtered using the shortest distance to surfaces function, and the volume fraction of C3d inside GFAP to GFAP and CD68 inside IBA1 to IBA1 were then quantified. The fluorescence intensity of PV and GAD65/67 were measured by Fiji software. The process was performed for three slices per mouse, with a total of five mice per group. Average data were used to represent the data for each mouse.

### 2.6 Immunohistochemistry (IHC)

The brain slices were obtained as described in IF, and a One‐step immunohistochemical kit (KGOS300, KeyGen Bio‐Tech) was used for IHC staining. Briefly, the slices were rinsed for 3 × 5 min with PBS and soaked in 3% H_2_O_2_‐methanol solution for 10 min to eliminate the interference of endogenous catalase. The slices were rinsed 3 × 5 min with PBS and blocked with 5–10% goat serum containing 0.3% Triton X‐100 for 1 h at RT, and then incubated with the primary antibody of CD68 (1:500, 137001; BioLegend) overnight at 4°C. The slices were rinsed 3 × 5 min with PBS, and then incubated with solution B and solution C for 30 min, and rinsed 3 × 5 min with PBS between the incubation. The DAB was used to visualize the CD68 signals. The images were captured by Olympus microscope BX43, and the CD68‐positive cells were analyzed by a blind experimenter using Fiji software. Three slices per mouse and five mice per group were sampled. Average data were used to represent the data for each mouse.

### 2.7 Western blot (WB)

Mice were sacrificed after being anesthetized with 5% isoflurane, and then the hippocampal tissues were collected on an ice plate. The tissues were homogenized with RIPA‐containing proteinase inhibitor and phosphatase inhibitor and PMSF, and then continued lysed on the wheel at 4°C for 30 min. The homogenates were centrifuged at 9391 *g* for 15 min at 4°C, and then the supernatants were collected. The BCA assay method was used to quantify the concentration of total protein and 1 μg/μL protein samples were prepared with protein loading buffers. Each protein sample (20 μg) was separated by SDS/PAGE and then transferred to PVDF membranes. The membranes were blocked in 5% nonfat dry milk at RT for 1 h, and then incubated primary antibodies including CD68 (1:1000, 137001; BioLegend), IBA1(1:1000, ab178846; Abcam), GLT‐1 (1:4000, ab41621; Abcam), PSD95 (1:2000, MABN68; Millipore), GluA1 (1:1000, ab31232; Abcam), GluA2 (1:1000, ab206293; Abcam), and GAPDH (1:2000, ab8245; Abcam) at 4°C overnight. The membranes were rinsed 3 × 5 min with TBST and then incubated with HRP‐conjugated secondary antibodies according to primary antibodies at RT for 1 h. The membranes were rinsed 3 × 5 min with TBST and developed by electrogenerated chemiluminescence solutions. The protein expression level was quantified using Fiji software.

### 2.8 Real‐time quantitative polymerase chain reaction (RT‐qPCR)

Trizol reagent (Invitrogen) was used to extract total RNA from mouse hippocampal tissues. 1 μg total RNA was used to reverse transcribed using HiScript III RT SuperMix for qPCR (Vazyme Biotech, R323‐01). The obtained cDNA was mixed with gene‐specific primers (Table S1) and ChamQ Universal SYBR qPCR Master Mix (Q711‐02; Vazyme Biotech) for RT‐qPCR in light cycler 480 instruments (Roche). The cycle time values were standardized to GAPDH of the same sample, and 2^−ΔΔCT^ was used to represent relative quantity.

### 2.9 Enzyme‐linked immunosorbent assay (ELISA)

Hippocampus tissues were harvested and the supernatants in homogenates were prepared as described above. The level of TNF‐α, IL‐1α, and C1q was determined using the TNF‐α ELISA kit (BMS607‐3; Thermo Fisher), IL‐1α (BMS611; Thermo Fisher) ELISA kit, and C1q ELISA kit (MBS2702391; MyBioSource) according to manufacturer's instruction.

### 2.10 Golgi staining

Mice were sacrificed after being anesthetized with 5% isoflurane, and the whole brain rapidly was harvested and then rinsed with ddH_2_O to remove blood. The frontal cortex and cerebellum brain were removed and the whole hippocampus was used for Golgi Staining (PK‐401; FD Neurotechnologies, Columbia, MD, USA). The pyramidal neurons and their spines in the CA1 region were photographed by microscope (Olympus) with 20× and 100× objective lenses, respectively. Fiji software with Neuro J and Sholl analysis plug‐ins was used to analyze the neuronal morphology including total dendritic length, branching points, and intersections as well as dendritic spines.

### 2.11 Microdialysis and High‐Performance Liquid Chromatography (HPLC)

Microdialysis probes (the length of 2 mm) were inserted under the guide at 24 h after myocardial IR. The hippocampal CA1 region was perfused with ACSF (140 mM NaCl, 3.0 mM KCl, 1.2–3.4 mM CaCl_2_, 1.0 mM MgCl_2_, 1.2 mm Na_2_HPO_4_, 0.27 mm NaH_2_PO_4_, 7.2 mm glucose, pH 7.4) by Eicom microdialysis system at 1 μL/min. Three samples were collected from freely moving mice at 30 min intervals after a 30 min washout period which aim to prevent blood contamination. All dialysates were stored immediately at −80°C until used.

The HPLC analysis was done accordingly (Luna‐Munguía et al., 2012). Briefly, 10 μL dialysate was automatically derivatized with 20 μL OPA working solution (70 mg OPA, 1 mL methanol, 95 mL boric acid buffer), and then 20 μL derivatized sample was injected into Agilent 1260 Infinity II HPLC system (Agilent, USA) and detected with a UV detection (334 nm) after 1 min. An Ultimate XB‐C18 chromatographic column (Yuexu Technology Inc, Shanghai) was used and maintained at 35°C. A gradient program of two mobile phases was applied at 1 mL/min. Solution A was a 90/10 (vol/vol) mixture of 50 mM sodium acetate and methanol, and the pH was adjusted to 5.75 by glacial acetic acid. Solution B was a 20/80 (vol/vol) mixture of solution A and methanol, and the pH was adjusted to 6.75 by glacial acetic acid. The glutamate levels were quantified against standard solutions.

### 2.12 Electrophysiology recording

#### 2.12.1 In vivo electrophysiology recording

The local field potentials (LFP) of neurons in the hippocampal CA1 region were collected by Apollo neural signal acquisition system (Kedou Brain‐computer Technology Co., Ltd, Suzhou, China). During the process, keep a quiet environment and reduce the noise signals as much as possible. The signals were filtered with a pass‐band of 0.3–200 Hz and were further amplified and digitized at 2 kHz. Neuro Explore 5 software (Plexon Inc., Dallas, TX) was performed to analyze the collected LFP signals, mainly including theta (3–8 Hz) and gamma (30–100 Hz) bands' power.

#### 2.12.2 Acute brain slices electrophysiology recording

Acute transverse hippocampal slices from 6 to 8 weeks of male C56BL/6 mice were made as described previously (Wang et al., 2016). Briefly, mice were anesthetized with 5% isoflurane and transcardially perfused with 15 mL cold and 95% O_2_/5% CO_2_ saturated high sucrose cutting solution (212.7 mM sucrose, 2.6 mM KCl, 1.23 mM NaH_2_PO_4_, 26 mM NaHCO_3_, 10 mM dextrose, 3 mM MgCl_2_, and 1 mM CaCl_2_), respectively. After perfusion, the whole brain was collected immediately. Transverse slices (350 μm) including the hippocampus were cut by using a vibratome (Leica VT 1000s) in a 95% O_2_/5% CO_2_ saturated high sucrose cutting solution. Then placed the slices into an incubation chamber containing 95% O_2_/5% CO_2_ saturated ACSF (124 mM NaCl, 5 mM KCl, 1.25 mM NaH_2_PO_4_, 26 mM NaHCO_3_, 10 mM dextrose, 1.5 mM MgCl_2_, and 2.5 mM CaCl_2_). The slices were recovered at 30°C and room temperature for 30 min, respectively. The voltage clamp was used to record NMDAR tonic currents of pyramidal neurons and PV interneurons, and miniature excitatory postsynaptic currents (mEPSCs) and miniature inhibitory postsynaptic currents (mIPSCs) of pyramidal neurons. All recordings were done in a submersion chamber perfused with 95% O_2_/5% CO_2_ saturated ACSF at 2 mL/min. For the NMDAR currents recording, the voltage clamp potential was +40 mV, 4–6 Ω borosilicate glass microelectrode filled the internal solution consisting of 115 mM CsMeSO_4_, 20 mM CsCl, 10 mM HEPES, 2.5 mM MgCl_2_.6H_2_O, 4 mM Mg‐ATP, 0.4 mM Na_3_‐GTP, 0.6 mM EGTA, 5 mM QX314, 0.1 mM Spermine‐Cl_4_, and PH was adjusted to 7.2 with CsOH.1 μM TTX, 100 μM picrotoxin, 10 μM bicuculline, and 10 μM DNQX were used to block action potentials, gamma‐aminobutyric acid (GABA) and α‐amino‐3‐hydroxy‐5‐methyl‐4‐isoxazole propionic acid receptor (AMPAR) currents. After baseline stability, 100 μM APV was added to get NMDAR tonic currents. For the mEPSCs recording, the voltage clam potential was −70 mV. 1 μM TTX, 100 μM picrotoxin, and 10 μM bicuculline were used to block action potentials and GABA currents. For mIPSCs recording, the voltage clam potential was −70 mV. The internal solution consisted of 70 mM CsMeSO_4_, 70 mM CsCl, 10 mM HEPES, 8 mM NaCl, 4 mM Mg‐ATP, 0.3 mM Na_3_‐GTP, 0.3 mM EGTA, and the PH was adjusted to 7.3–7.4 with CsOH. 1 μM TTX, 100 μM APV, and 10 μM DNQX were used to block action potentials, NMDAR, and AMPAR currents. All recordings were collected with a 700B amplifier (Axon Instrument, Foster City), filtered at 2 kHz, and digitized at 10 kHz. One neuron per slice and six to eight slices from three to four mice per group were sampled.

### 2.13 Statistical analyses

Data were presented as mean ± SEM and analyzed with two‐way ANOVA followed by Bonferroni post hoc for multiple comparisons or an unpaired t‐test when appropriate (SPSS 25.0, IBM, NY, USA). The figures were created with GraphPad Prism 9.0 (GraphPad Software, CA, USA). The *F*‐values, degrees of freedom, and *p*‐values for the interaction, row factor, and column factor, as analyzed using a two‐way ANOVA, are presented in Table S2. A *p*‐value <0.05 was set for statistical significance.

## 3 RESULTS

### 3.1 Cardiac surgery‐induced delirium‐like behavior in aged mice

**FIGURE 1:**
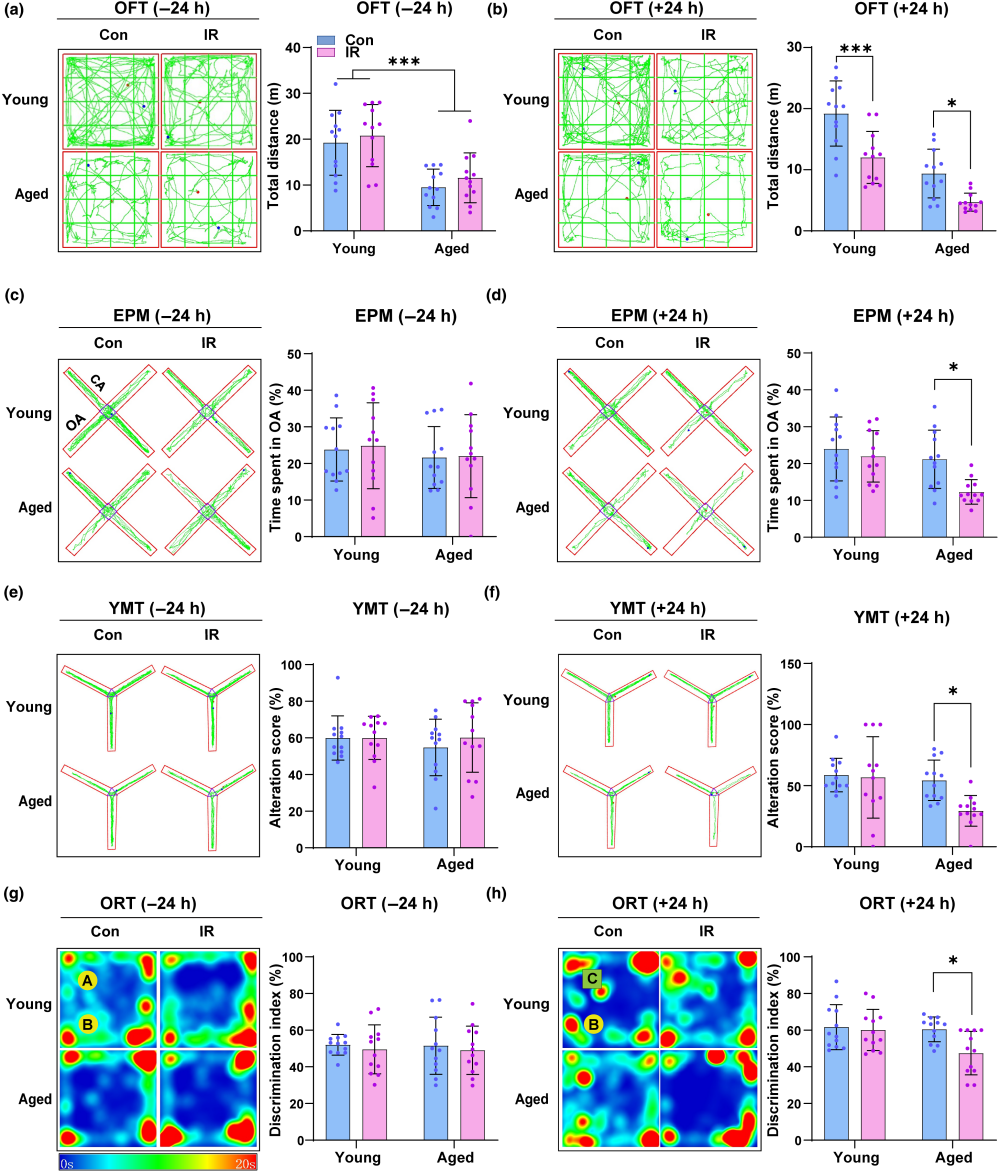
Cardiac surgery induces delirium‐like behavior in aged mice. (a–h) Representative images or heatmap of the path of one animal in open‐filed test (OFT), elevated plus maze (EPM), Y maze test (YMT), and object recognition test (ORT). (a, b) Motion ability was assessed by measuring the total distance with the OFT 24 h before cardiac surgery or 24 h after cardiac surgery. (c, d) Anxiety‐like behavior was evaluated by measuring the time spent in OA with the EPM 24 h before cardiac surgery or 24 h after cardiac surgery. (e, f) Spatial memory was evaluated by measuring alteration score with the YMT 24 h before cardiac surgery or 24 h after cardiac surgery. (g, h) Cognition memory was evaluated by measuring the discrimination index with the ORT 24 h before cardiac surgery or 24 h after cardiac surgery. Data are expressed as mean ± SD (*n* = 12 per group). Two‐way ANOVA, followed by Bonferroni post hoc for multiple comparisons, was used to analyze the data. *p < 0.05, ***p < 0.001.

The effects of age and cardiac surgery on delirium‐like behavior were assessed in the OFT, YMT, EPM, and ORT at 24 h after open‐chest myocardial IR. To eliminate the potential contribution of baseline differences in activity and cognition on delirium‐like behavioral assessments, all the selected behavioral tests were first performed 24 h before surgery. The results showed the total distance in the OFT was decreased in the aged group compared with the young group (*p* < 0.0001, *t* = 5.572, df = 46) (Figure 1a), whereas there was no significant difference in the percentage of time spent in open arm (OA) in the EPM (*p* = 0.3875, *t* = 0.8725, df = 46) (Figure 1c), alteration score in YMT (*p* = 0.5638, *t* = 0.5814, df = 46) (Figure 1e), and discrimination index in the ORT (*p* = 0.8938, *t* = 0.1343, df = 46) (Figure 1g) between the young group and aged group. Then the behavioral tests were repeated at 24 h after cardiac surgery, and surgery was found to significantly decrease the total distance of both young (*p* = 0.0004, *t* = 4.372, df = 44) and aged mice (*p* = 0.0394, *t* = 2.854, df = 44) compared with the control group (Figure 1b), while the percentage of time spent in OA (*p* = 0.0207, *t* = 3.091, df = 44) (Figure 1d), alteration score (*p* = 0.0313, *t* = 2.939, df = 44) (Figure 1f), and discrimination index (*p* = 0.0247, *t* = 4.182, df = 44) (Figure 1h) were compromised only in the aged mice. These data suggest that both aging and/or cardiac surgery impaired their movement ability, and aging significantly increased the susceptibility to developing delirium‐like behavior following surgery.

### 3.2 Increased A1 astrocyte activation is closely associated with the occurrence of delirium‐like behavior

**FIGURE 2:**
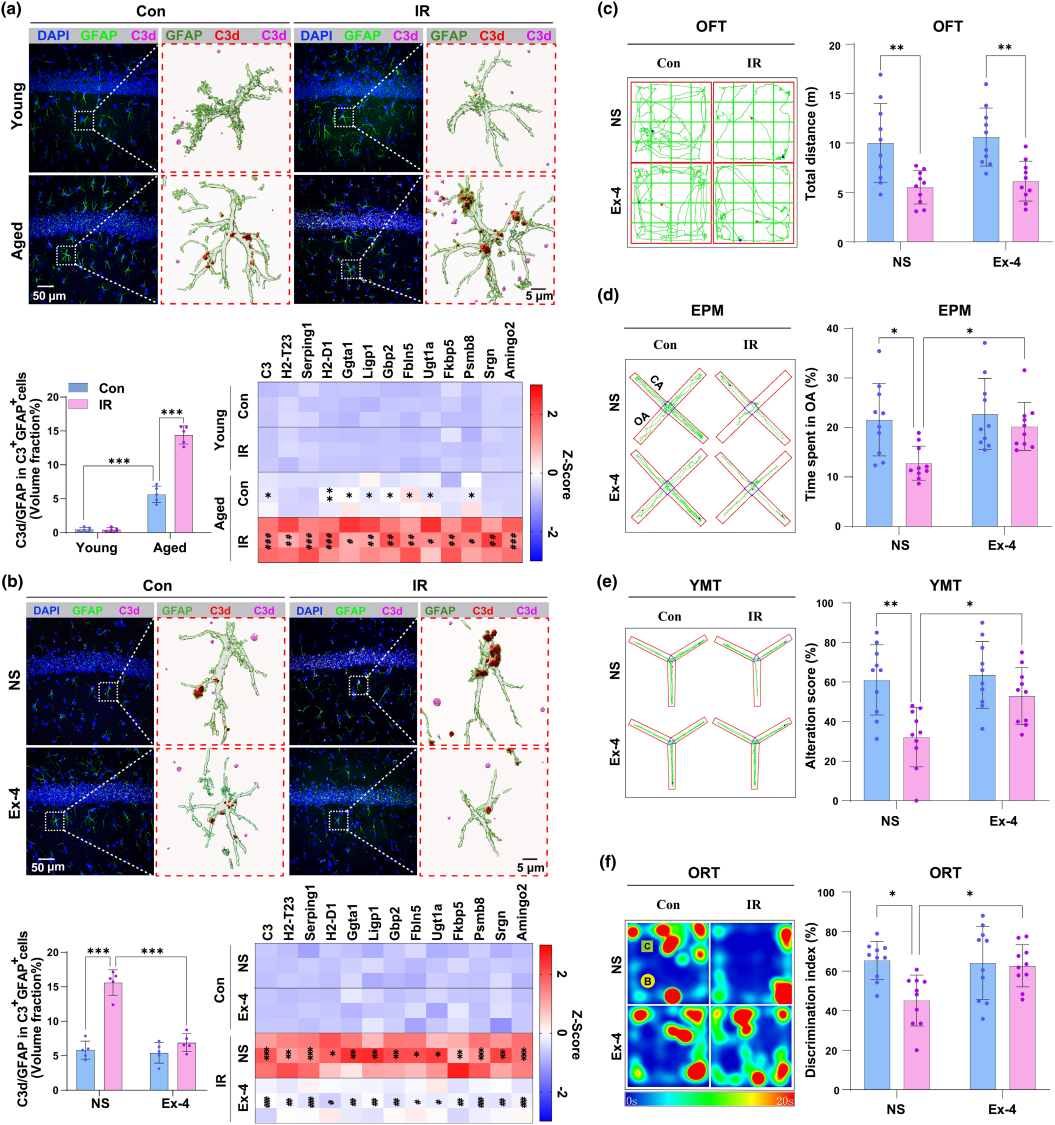
Cardiac surgery leads to a significant increase in hippocampal A1 astrocytes and delirium‐like behaviors in aged mice, and injection of exendin‐4 (Ex‐4) can reverse the alterations. (a, b) Representative confocal images of GFAP (Green), C3d (Magenta), and DAPI (Blue) and 3D rendering of GFAP (Cyan), C3d inside GFAP (Red), and C3d outside GFAP (Magenta). The activation level of A1 astrocytes was evaluated by measuring the volume fraction of C3d inside GFAP to GFAP, and the expression levels of A1‐special transcripts in the hippocampus of young and aged mice by immunofluorescence (IF) and real‐time quantitative polymerase chain reaction (RT‐qPCR). Both young and aged mice were used in (a) as indicated, but only aged mice were used in (b–f). (c–f), Representative images or heatmap of the path of one animal in OFT, EPM, YMT, and ORT. The effect of NS or Ex‐4 on motion function (c), anxiety‐like behavior (d), spatial memory (e), and discrimination memory (f) of aged mice undergoing cardiac surgery was assessed by measuring the total distance with OFT, time spent in OA with EPM, alteration score with YMT and discrimination index with ORT, respectively. Data are expressed as mean ± SD (*n* = 5 per group for IF, *n* = 3 per group for RT‐qPCR, and *n* = 10 per group for behavioral tests). Two‐way ANOVA, followed by Bonferroni post hoc for multiple comparisons, was used to analyze the data. *p < 0.05, **p < 0.01, ***p < 0.001. For heatmap in (a), *p < 0.05 and **p < 0.01 versus the Young + Con group; ^#^ *p* < 0.05, ^##^ *p* < 0.01, and ^###^ *p* < 0.001 versus the Aged + Con group. For heatmap in (b), *p < 0.05, **p < 0.01, and ***p < 0.001 versus the Con + NS group; ^#^ *p* < 0.05, ^##^ *p* < 0.01, and ^###^ *p* < 0.001 versus the IR + NS group.

First, we used immunofluorescence (IF) and RT‐qPCR to investigate the immune active changes of A1 astrocytes after cardiac surgery in the young and aged mice by detecting astrocytes marker GFAP and A1 astrocytes marker C3d, and A1‐specific mRNA (C3, H2–T23, Serping1, H2–D1, Ggta1, Ligp1, Gbp2, Fbln5, Ugt1a, Fkbp5, Psmb8, Srgn, and Amigo2) in the hippocampus as previous studies (Clarke et al., 2018; Fang et al., 2022; Li et al., 2022). Compared with the Young + Control (Con) mice, the volume fraction of C3d inside GFAP to GFAP (*p* < 0.001, *t* = 8.788, df = 16) (Figure 2a) and some A1 astrocytes mRNA (*p* < 0.05 in C3, Ggta1, Ligp1, Gbp2, Fbln5, Ugt1a, Psmb8; *p* < 0.01 in H2‐D1) (Figure 2a) were increased in the Aged + Con mice. Compared with the control mice, cardiac surgery only induced a significant increase in the volume fraction of C3d inside GFAP to GFAP (*p* < 0.001, *t* = 14.96, df = 16) (Figure 2a) and in all A1‐specific mRNA (*p* < 0.05 in Ggta1, Ugt1a and Psmb8; *p* < 0.01 in H2‐T23, Ligp1, Gbp2, Fbln5, Fkbp5, Srgn; *p* < 0.001 in other markers) (Figure 2a) in the aged mice, but no alteration in the young mice. The representative *Z*‐stack images and the 3D reconstruction video created using Imaris, showcasing GFAP, C3d, and DAPI, are presented in Video S1. To prevent the risk of false positive signals in C3d detection, attributed to the non‐specific binding of the fluorescent secondary antibody, we implemented a negative control test for the experiments (Figure S3).

**FIGURE 3:**
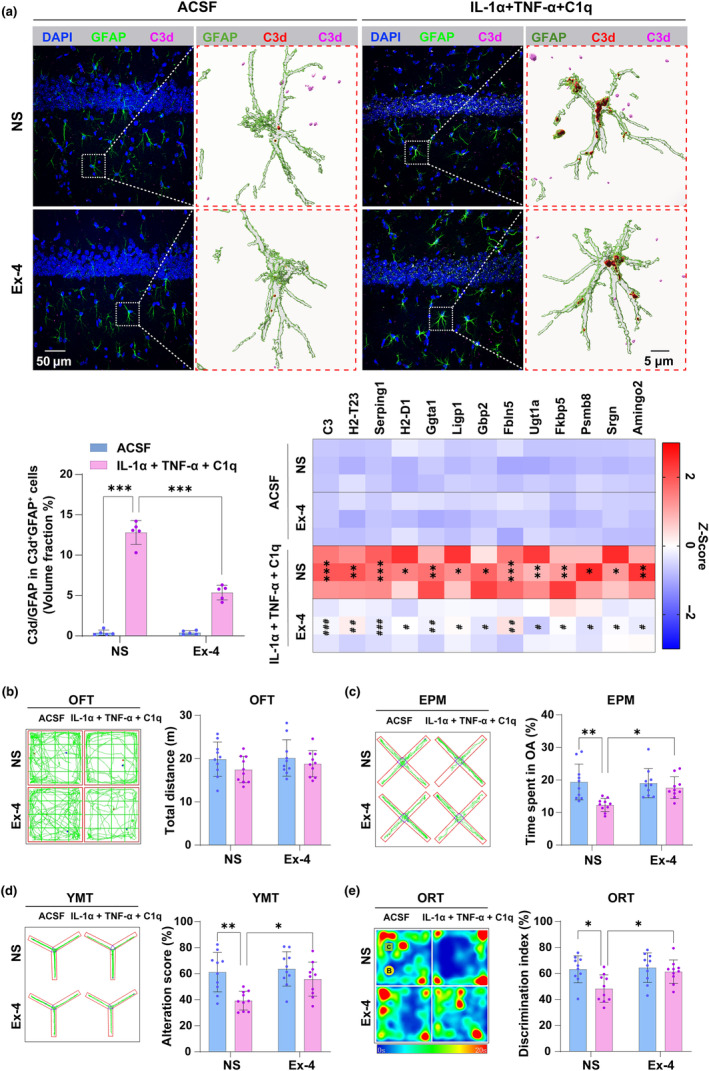
IL‐1α, TNF‐α and C1q induce delirium‐like behavior in young mice through upregulation of A1 astrocytes. (a) Representative confocal images of GFAP (Green), C3d (Magenta) and DAPI (Blue), and 3D rendering of GFAP (Cyan), C3d inside GFAP (Red), and C3d outside GFAP (Magenta). The effect of i.c.v. injection of ACSF or IL‐1α, TNF‐α, and C1q following with intraperitoneal administration of NS or Ex‐4 on A1 astrocytes in the hippocampus of young mice was evaluated by measuring the volume fraction of C3d inside GFAP to GFAP, and the expression levels of A1‐special transcripts in the hippocampus of young mice undergoing i.c.v. injection of ACSF or IL‐1α, TNF‐α, and C1q following with intraperitoneal administration of NS or Ex‐4 were evaluated by IF and RT‐qPCR. (b‐e) Representative images or heatmap of the path of one animal in OFT, EPM, YMT, and ORT. The effect of i.c.v. injection of ACSF or IL‐1α, TNF‐α, and C1q following with intraperitoneal administration of NS or Ex‐4 on motion function (b), anxiety‐like behavior (c), spatial memory (d), and discrimination memory (e) of young mice undergoing cardiac surgery was assessed by measuring the total distance with OFT, time spent in OA with EPM, alteration score with YMT and discrimination index with ORT, respectively. Data are expressed as mean ± SD (*n* = 5 per group for immunofluorescence, *n* = 3 per group for RT‐qPCR, and *n* = 10 per group for behavioral tests). Two‐way ANOVA, followed by Bonferroni post hoc for multiple comparisons, was used to analyze the data. *p < 0.05, **p < 0.01, and ***p < 0.001. For heatmap in (a), *p < 0.05, **p < 0.01, and ***p < 0.001 versus the ACSF + NS group; ^#^ *p* < 0.05, ^##^ *p* < 0.01, and ^###^ *p* < 0.001 versus the IL‐1α + TNF‐α + C1q + NS group.

Second, we explored the effect of downregulation or upregulation of A1 astrocyte activation on delirium‐like behavior in aged or young mice, respectively. The results showed that intraperitoneal injection of exendin‐4 (Ex‐4, 300 ng/kg) (Shan et al., 2019) immediately after cardiac surgery significantly decreased the immunoactivity of A1 astrocytes (Figure 2b) and simultaneously ameliorated delirium‐like behavior induced by surgery (Figure 2c–f) in the aged mice. Whereas, i.c.v. administration of IL‐1α, TNF‐α, and C1q can increase their immunoactivity and mimic delirium‐like behavior in the young mice, and intraperitoneal injection of Ex‐4 attenuated such changes (Figure 3a–e).

These results suggested that the increased immunoactivity of A1 astrocytes in the hippocampus was closely involved in the delirium‐like behavioral development in aged mice after surgery.

### 3.3 Cardiac surgery increased microglia activation and release of IL‐1α, TNF‐α, and C1q

Microglia activation was reported to induce A1 astrocytes phenotypic changes through IL‐1α, TNF‐α, and C1q release (Liddelow et al., 2017). We then explored the adverse effects of age and cardiac surgery on microglia. Microglia marker IBA1 and microglia activation marker CD68 were detected by IF, immunohistochemical (IHC), and western blot, and similar findings to A1 astrocytes were found. The volume fraction of CD68 inside IBA1 to IBA1 (*p* < 0.001, *t* = 8.289, df = 16) (Figure S1A), the CD68‐positive cells (*p* < 0.001, *t* = 7.027, df = 16) (Figure S1B), and the expressions of CD68 (*p* < 0.001, *t* = 11.18, df = 8) and IBA1 (*p* = 0.0034, *t* = 5.499, df = 8) (Figure S1C) were only increased in aged mice after cardiac surgery. In addition, aged mice had a higher basal level of IL‐1α (*p* = 0.0399, *t* = 3.274, df = 12), TNF‐α (*p* = 0.0336, *t* = 3.367, df = 12), and C1q (*p* = 0.0422, *t* = 3.244, df = 12) in the hippocampus compared with the young mice (Figure S1D), and surgery further aggravated the release of these inflammatory mediators (IL‐1α: *p* = 0.006, *t* = 4.322, df = 12; TNF‐α: *p* = 0.013, *t* = 3.887, df = 12; C1q: *p* = 0.0456, *t* = 3.202, df = 12) (Figure S1D). These results suggested the activation of microglia and the releasing of IL‐1α, TNF‐α, and C1q are closely involved in the increase of A1 astrocyte activation. The representative *Z*‐stack images and the 3D reconstruction video, created using Imaris for IBA1, CD68, and DAPI, are presented in Video S2. To prevent the risk of false positive signals in CD68 detection attributed to non‐specific binding of the fluorescent secondary antibody, we also do a negative control test for the experiments (Figure S4)

### 3.4 Cardiac surgery led to the excessive accumulation of glutamate in the extrasynaptic space via downregulating GLT‐1 expression

**FIGURE 4:**
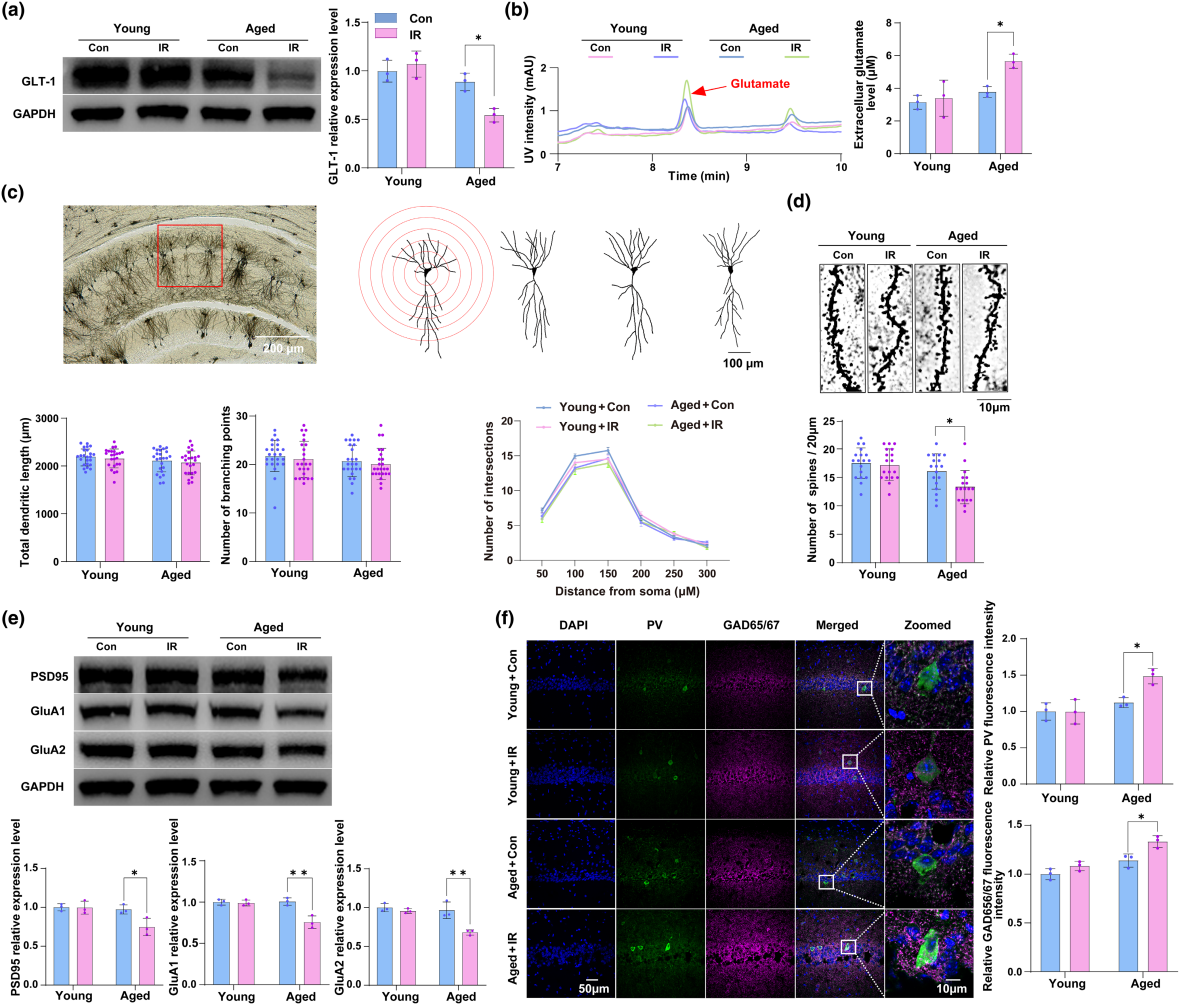
Cardiac surgery leads to the excessive accumulation of glutamate in extrasynaptic space via downregulation of GLT‐1, and then decreases the excitability of pyramidal neurons but increases the inhibition of PV interneurons in the hippocampus. (a) The GLT‐1 protein expression level was evaluated by WB. (b) The level of glutamate in the extrasynaptic space was detected with microdialysis and HPLC. (c, d) The morphology of pyramidal neurons including total dendritic length, the number of branching points, the number of intersections, and the dendritic spines of pyramidal neurons were evaluated by Golgi staining. (e) The expression levels of excitatory synapse protein PSD95, GluA1 and GluA2 in the hippocampus after cardiac surgery were assessed by WB. (f) The expression levels of PV and GAD65/67 which represent the inhibitability of PV interneurons after cardiac surgery were assessed by IF. Data are expressed as mean ± SD (*n* = 3 per group, and 8 neurons per mouse for Golgi staining, *n* = 3 per group for WB or IF). Two‐way ANOVA, followed by Bonferroni post hoc for multiple comparisons, was used to analyze the data. *p < 0.05, **p < 0.01.

It has been reported the GLT‐1 expression and the ability to clear glutamate of A1 astrocytes were decreased with aging (Li et al., 2019). We, therefore, investigated whether GLT‐1 expression level was downregulated and glutamate was excessively accumulated in the extrasynaptic space in mice after surgery. The GLT‐1 expression was significantly decreased (*p* = 0.0232, *t* = 4.014, df = 8) (Figure 4a), and the glutamate level was significantly increased (*p* = 0.046, *t* = 3.536, df = 8) (Figure 4b) in the aged mice after surgery, whereas no changes were detected in the young mice. The retention time of glutamate in the chromatographic column was determined using a 50 μM standard of glutamate, as shown in Figure S2. These data suggested that increased A1 astrocyte activation induced by cardiac surgery led to glutamate accumulation in the extrasynaptic space in the aged mice.

### 3.5 The excessive glutamate accumulation decreased the excitability of pyramidal neurons but increased the PV interneurons inhibition in the hippocampus

Pyramidal neurons and PV interneurons are indispensable excitatory and inhibitory neurons in the hippocampus, respectively, and both play a critical role in cognitive performance (Verret et al., 2012; Wu et al., 2002). Given the excessive accumulation of glutamate altered the activities of neurons, we assumed that the activities of pyramidal neurons and PV interneurons may be changed in the aged mice after cardiac surgery. The Golgi staining was used to analyze the morphology of pyramidal neurons and the western blot was applied to detect the expression levels of excitatory synaptic proteins such as PSD95, GluA1, and GluA2. The results showed the number of spines (*p* = 0.0351, *t* = 2.846, df = 68) (Figure 4d) and expression levels of PSD95 (*p* = 0.0482, *t* = 3.505, df = 8), GluA1 (*p* = 0.002, *t* = 5.986, df = 8), and GluA2 (*p* = 0.0035, *t* = 5.489, df = 8) (Figure 4e) were significantly decreased in the aged but not young mice after cardiac surgery, which suggested the decrease in excitability of neurons. However, no difference was found in the total dendritic length, the number of branching points, and intersections of pyramidal neurons in the hippocampal CA1 region among groups (Figure 4c). IF was used to assess the expression levels of PV and GAD65/67 (a key enzyme for GABA synthesis), the high expression levels of them were usually regarded as an increased inhibition of PV interneurons. The expression levels of PV (*p* = 0.0378, *t* = 3.671, df = 8) and GAD65/67 (*p* = 0.0228, *t* = 4.028, df = 8) (Figure 4f) were increased in the aged mice but not in the young mice after cardiac surgery. The above data indicated that the excitability of pyramidal neurons was decreased, while the inhibition of PV interneurons was increased in the aged mice after cardiac surgery.

### 3.6 Enhanced tonic activation of NMDAR was responsible for the abnormal activities of pyramidal neurons and PV interneurons in the hippocampus

**FIGURE 5:**
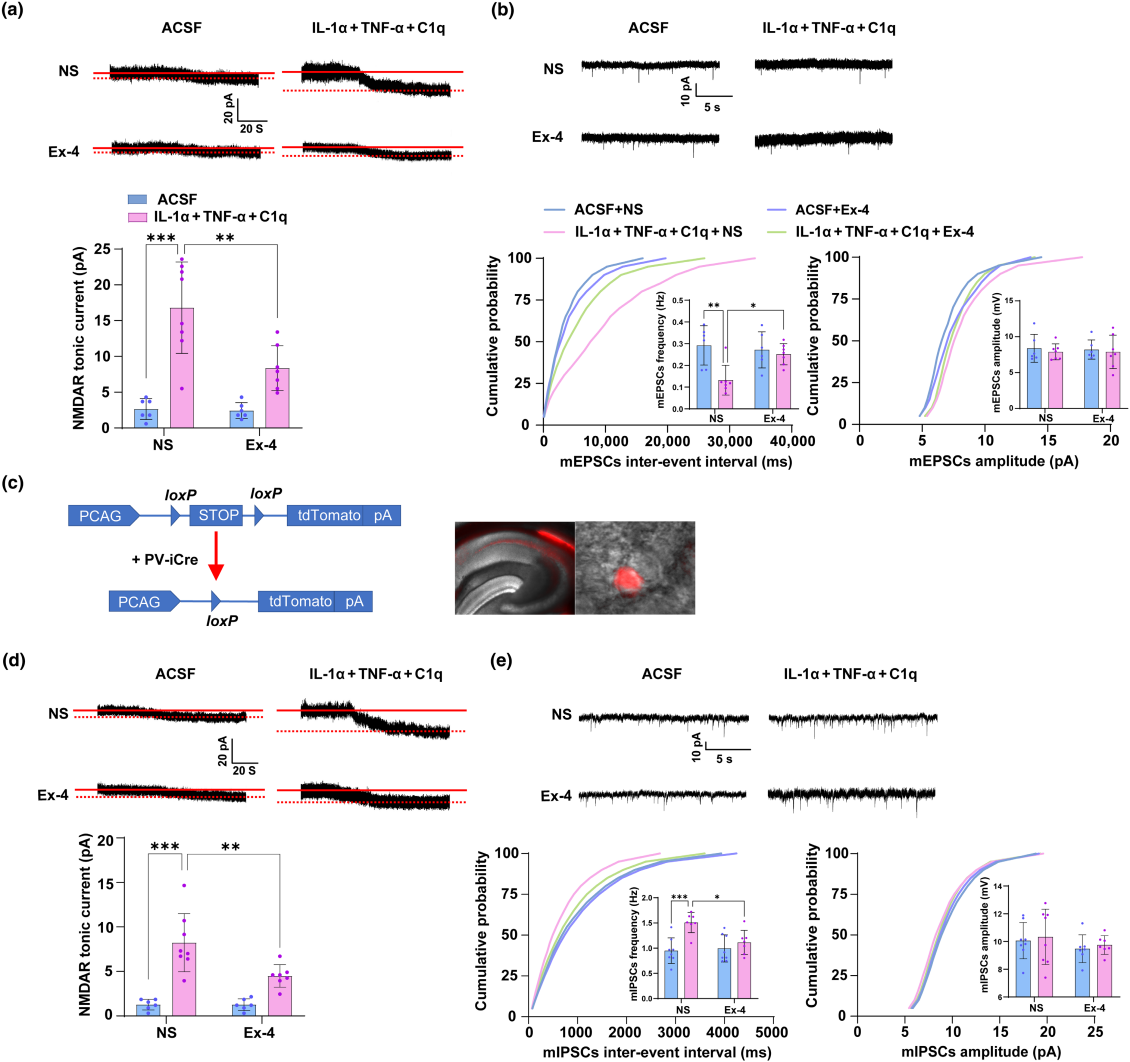
Enhanced tonic activation of the NMDAR is responsible for the abnormal activities of pyramidal neurons and PV interneurons in the hippocampus. (a, b) The NMDAR tonic currents (a) and mEPSCs (b) of pyramidal neurons in the CA1 region after i.c.v. injection of ACSF or IL‐1α, TNF‐α, and C1q following with intraperitoneal administration of NS or Ex‐4 were detected by patch‐clamp. (c) The strategy to obtain tdTomato positive PV interneurons mice. (d, e) The NMDAR tonic currents of PV interneurons (d) and mIPSCs (e) of pyramidal neurons in the CA1 region after i.c.v. injection of ACSF or IL‐1α, TNF‐α, and C1q following with intraperitoneal administration of NS or Ex‐4 were detected by patch‐clamp. Data are expressed as mean ± SD (6 to 8 slices from 3 to 4 mice per group). Two‐way ANOVA, followed by Bonferroni post hoc for multiple comparisons, was used to analyze the data. *p < 0.05, **p < 0.01, and ***p < 0.001.

Previous studies reported that the massive accumulation of glutamate in the extrasynaptic space enhanced the tonic activation of the NMDAR and regulated the activities of pyramidal neurons and PV interneurons (Hanson et al., 2019; Miller et al., 2014; Talantova et al., 2013). Thus, we speculated that the alterations of the activities of pyramidal neurons and PV interneurons after cardiac surgery may be induced through the enhanced tonic activation of NMDAR. To investigate this, we induced A1 astrocyte activation in the hippocampus in the young mice by i.c.v. injection of IL‐1α, TNF‐α, and C1q, and reversed their upregulation by intraperitoneal injection of Ex‐4. Then the NMDAR tonic currents were recorded from pyramidal neurons and PV interneurons, respectively, and recorded mEPSCs and mIPSCs were from pyramidal neurons. The results showed IL‐1α, TNF‐α, and C1q application significantly enhanced NMDAR tonic currents of the pyramidal neurons (*p <* 0.0001, *t* = 6.61, df = 23) (Figure 5a) and PV interneurons (*p <* 0.0001, *t* = 6.579, df = 23) (Figure 5d), decreased the mEPSCs frequency (*p =* 0.0044, *t* = 3.924, df = 22) (Figure 5b), and increased the mIPSCs frequency (*p =* 0.0009, *t* = 4.434, df = 26) (Figure 5e). The administration of Ex‐4 mitigated the above changes. However, there were no changes in the amplitude of mEPSCs and mIPSCs in both neurons. These data indicated that induced A1 astrocyte activation led to the excitation/inhibition imbalance of hippocampal neurons via enhancing NMDAR tonic activation.

### 3.7 Cardiac surgery led to the abnormal activities of hippocampal neural networks in the aged mice

**FIGURE 6:**
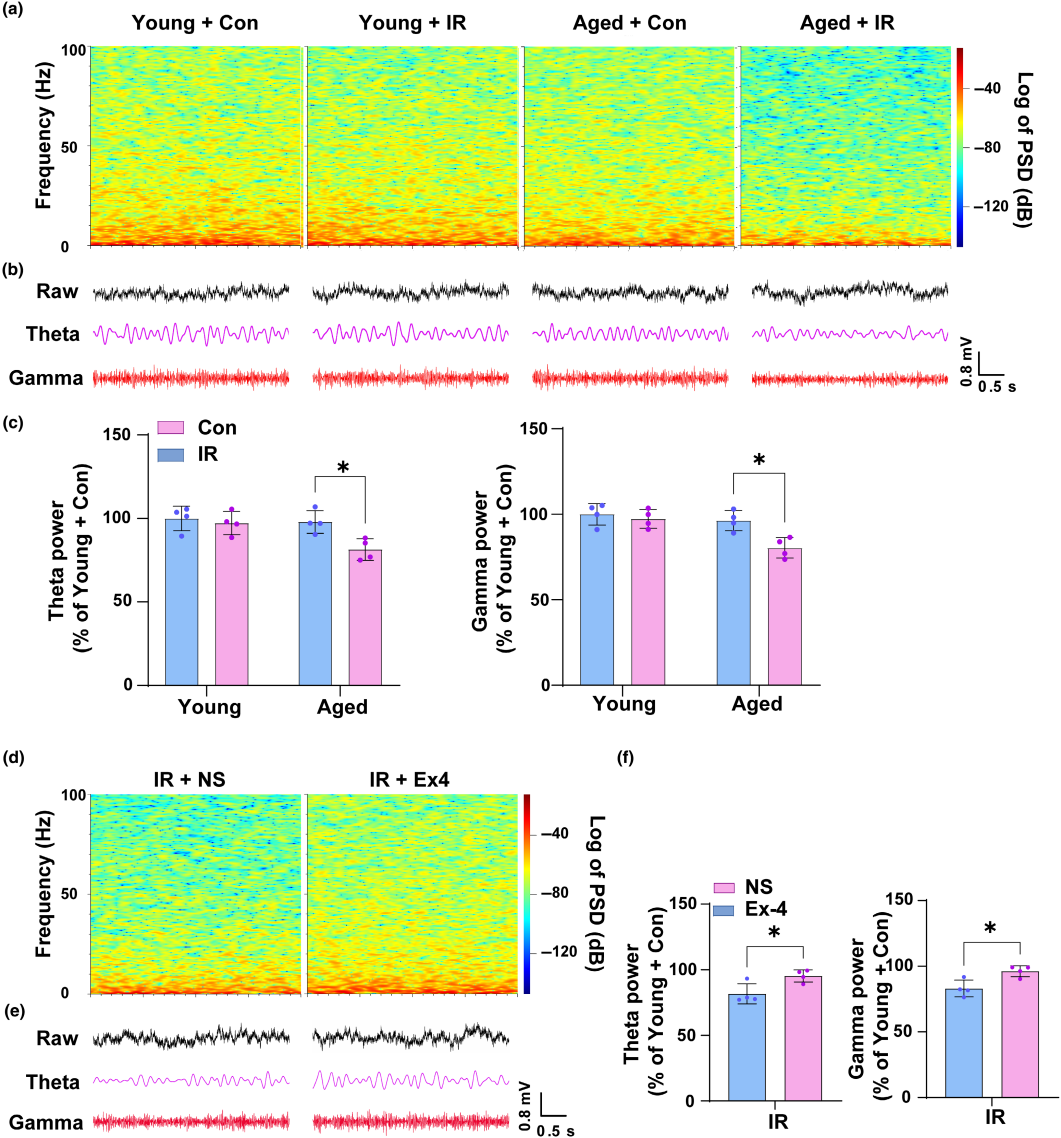
Cardiac surgery leads to abnormal hippocampal network activities in aged mice, and the alterations can be ameliorated by the administration of Ex‐4. (a) Representative images of the LFP power spectrum of the hippocampus in the indicated groups after cardiac surgery. (b) Raw data of LFP and filtered theta and gamma bands power of the hippocampus. (c) Relative quantification of theta and gamma bands power of the hippocampus. (d) Representative images of the LFP power spectrum of the hippocampus in aged mice after intraperitoneal injection of NS or Ex‐4 followed cardiac surgery. (e) Raw data of the LFP and filtered theta and gamma bands power of the hippocampus after intraperitoneal injection of NS or Ex‐4 followed cardiac surgery in aged mice. (f) Relative quantification of theta and gamma bands power of the hippocampus after intraperitoneal injection of NS or Ex‐4 followed cardiac surgery. Data are expressed as mean ± SD (*n* = 4 per group). Two‐way ANOVA, followed by Bonferroni post hoc for multiple comparisons, was used to analyze the data for (c); Unpaired *t*‐test was used to analyze the data for (f).*p < 0.05.

Previous studies found that theta and gamma oscillations were the most prominent rhythm recorded in the hippocampus in the awake state or REM sleep, and play a key role in the process of perception, attention, and memory (Buzsaki, 2002; Buzsaki & Draguhn, 2004; Colgin, 2016). Given the synchronization between the pyramidal neurons and PV interneurons is critical for the generation of theta and gamma oscillations (Bland et al., 2007; Buzsaki & Wang, 2012; Klausberger et al., 2003), we assumed that the theta and gamma oscillations may be changed in the aged mice after cardiac surgery. To test this, microfilament array electrodes were implanted into the hippocampal CA1 region, and the mice were allowed to recover for 7 days. Then myocardial IR was performed, and LFP was recorded 24 h later. The results showed that surgery significantly decreased the power of the theta (*p =* 0.0321, *t* = 3.392, df = 12) and gamma (*p =* 0.0152, *t* = 3.8, df = 12) oscillations in the aged but not young mice (Figure 6a–c). Inhibition of A1 astrocytes by administration of Ex‐4 reversed the theta (*p =* 0.0235, *t* = 3.018, df = 6) and gamma (*p =* 0.0139, *t* = 3.432, df = 6) oscillations' changes in aged mice undergoing cardiac surgery (Figure 6d–f). These data suggested that surgery led to the abnormal activities of the hippocampal neural networks in the aged mice, and the increased A1 astrocyte activation may be one of the major contributors to these phenomena.

## 4 DISCUSSION

**FIGURE 7:**
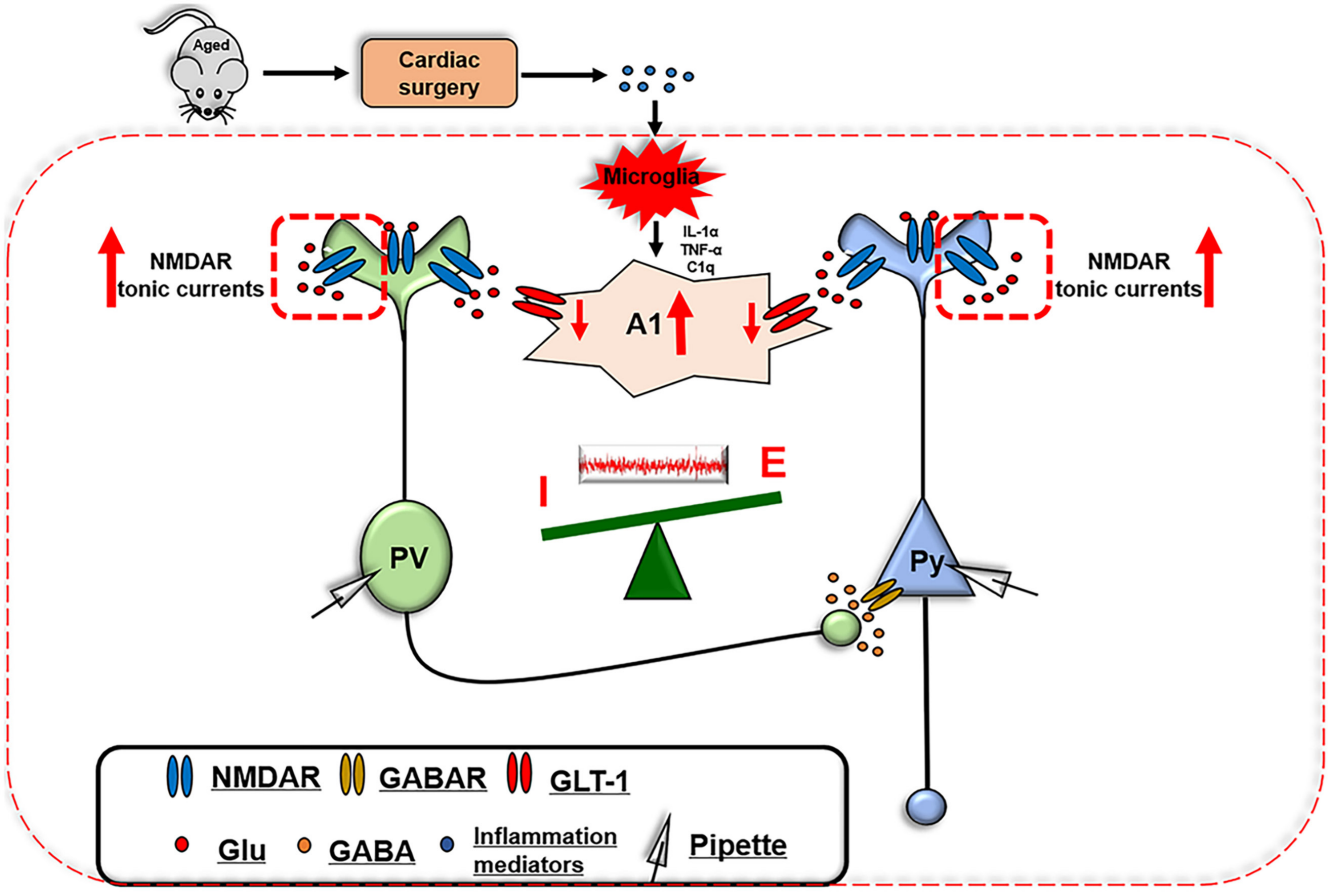
The general overview of the main highlights of this study. Cardiac surgery resulted in increased activation of microglia and A1 astrocytes, subsequently leading to an accumulation of glutamate. This accumulation impaired hippocampal neural networks by enhancing NMDAR tonic activation, thereby contributing to delirium‐like behavior in aged mice.

Our study revealed that both aging and A1 astrocyte activation induced by cardiac surgery contributed to postoperative delirium‐like behavioral development. The high level of A1 astrocyte activation in the hippocampus following surgery in the aged but not young mice subsequently resulted in excessive glutamate accumulation in the extrasynaptic space due to the GLT‐1 downregulation. All of these further enhanced the tonic activation of NMDAR in both the pyramidal neurons and PV interneurons which, in turn, induced the abnormality of the hippocampal neural networks. All these alterations ultimately mediated the occurrence of delirium‐like behavior (Figure 7).

Recently, a battery of behavioral tests was used to assess delirium‐like behavior in a mouse model which made preclinical studies of delirium possible (Cursano et al., 2021; Gile et al., 2018). Given that delirium predominantly occurs within the first 72 h after surgery, particularly during the initial 24 h, we thus decided to assess delirium‐like behavior 24 h post‐surgery (Chaiwat et al., 2019). This approach also aimed to minimize the direct effects of postoperative trauma on delirium‐like behavior immediately after the surgery. First, we assessed the locomotor activity of mice using OFT. In contrast to the previous studies (Peng et al., 2016; Zhang et al., 2018), our findings showed that the locomotion of both the young and aged mice was significantly impaired after surgery. The different surgery models may likely account for this discrepancy. Simple laparotomy was often used in previous delirium studies. Cardiac surgery may have a more significant impact on subjects compared to simple laparotomy. This is primarily due to the major trauma associated with cardiac procedures, which results in more severe tissue injuries. Consequently, the procedure led to higher inflammatory responses than those typically observed in abdominal surgeries, as previously reported. (Hovens et al., 2016).

Delirious patients often showed anxiety symptoms after surgery and we, therefore, used the EPM to detect the anxiety‐like symptoms of mice 24 h after cardiac surgery. In line with the previous studies (Cursano et al., 2021), aged but not young mice developed significant anxiety‐like symptoms 24 h after cardiac surgery in our study. Inattention and cognitive dysfunction are the main clinical manifestations of the POD and, hence, the well‐established behavioral tests of YMT and ORT were applied to assess both spatial and recognition memory in our study without unnecessary long‐term training which may have extra impacts on subjects (Kraeuter et al., 2019; Leger et al., 2013). Furthermore, both the YMT and the ORT may serve as indicators of the inherent attention and advanced neurological functions in mice. This is based on the well‐known concept that effective memory formation cannot occur without these higher order neurological functions. Our study found that both attention and memory were impaired only in the aged mice but not in young mice at 24 h after cardiac surgery. Our data likely suggested that aged mice are more susceptible to developing delirium‐like behavior at 24 h after cardiac surgery than young mice.

Astrocytes are widely distributed in the central nervous system and involved in various physiological or pathological processes in an age‐dependent manner. For example, normal aged mice were found to have highly activated A1 astrocytes in the hippocampus and striatum than young mice under LPS stimulation (Clarke et al., 2018). Another study found that in the aging brains of mice, astrocytes shifted toward a reactive phenotype. Concurrently, there was a general upregulation in the gene transcriptomes responsible for pruning synapses (Boisvert et al., 2018). All these may suggest astrocytes in aged brains may be in a partially and/or easily reactive state, which may well explain why the aging brain is more susceptible to insults during neurodegenerative disease development or postoperative delirium occurrence reported in the current study. GLP‐1R agonists are widely used in the treatment of diabetes and improve cognitive function by downregulating A1 astrocytes (Shan et al., 2019; Teramoto et al., 2011; Yun et al., 2018). Our study found that intraperitoneal administration of Ex‐4, a GLP‐1R agonist, ameliorated delirium‐like behavior in aged mice after cardiac surgery while i.c.v. injection of inflammatory mediators such as IL‐1α, TNF‐α, and C1q‐induced A1 astrocyte activation led to delirium‐like behavior in young mice.

Given that diabetes is a known risk factor for both cardiac diseases and POD (Kotfis et al., 2019), GLP‐1R agonists emerge as a promising treatment option. They may not only improve cardiac function post‐surgery, as previously reported (Ma et al., 2021; Nauck et al., 2017), but also potentially prevent the development of POD, as demonstrated in the current study. This is particularly relevant following cardiac surgery in diabetic and elderly patients. Additionally, increasing evidence indicates that the perioperative use of GLP‐1R agonists in cardiac surgery patients offers multiple benefits. These include maintaining optimal blood glucose levels, reducing myocardial damage, and inhibiting inflammatory response (Chen et al., 2016; Hulst et al., 2020). This may suggest that the perioperative use of GLP‐1R agonists in cardiac surgeries and beyond may offer some advantages over other drugs, notably in preventing delirium. Further clinical study is needed to verify its preventive effects on POD development.

It is noteworthy that some studies reported that GLP‐1R agonists improve neuronal function by directly activating GLP‐1R on neurons (Teramoto et al., 2011; Wang et al., 2021). Thus, Ex‐4 improving delirium‐like behavior in our study may be also mediated by its beneficial effect on neurons. Furthermore, GLP‐1R agonists were also reported to have a neuroprotective effect through direct inhibition of A1 astrocytes (Liddelow et al., 2017; Yun et al., 2018). Therefore, Ex‐4 protects against delirium‐like behavioral development likely through inhibiting A1 astrocytes and protecting neurons. Using conditional GLP‐1R knockout in neurons may well settle this argument and will be considered in our future study.

Pyramidal neurons are excitatory glutamatergic neurons and their spines are localized in their dendritic and constitute the postsynaptic structure of excitatory glutamatergic synapses (Ballesteros‐Yanez et al., 2006; Sorra & Harris, 2000). Synapses are highly plastic and manifested with the changes in the number of spines and the expression level of postsynaptic proteins such as PSD95, GluA1, and GluA2 (Diering & Huganir, 2018). It is well‐established that synaptic plasticity plays a critical role in the formation of memory (Martin et al., 2000). On the other hand, PV interneurons are inhibitory GABAergic neurons characterized by their PV‐positive and fast‐spiking phenotype (Dikmen et al., 2020). They are necessary for the control of pyramidal neuron output and rhythmic neuronal synchrony by releasing GABA, which promotes memory formation (Hu et al., 2014). Our data showed that the number of spines and the expression levels of PSD95, GluA1, and GluA2 were decreased, while the PV and GAD65/67 were increased in the hippocampus of the aged mice undergoing cardiac surgery. In addition, our study also showed that these alterations were caused by enhancing tonic activation of NMDAR in the pyramidal neurons and PV interneurons. These are likely the key molecular bases of delirium‐like behavior following surgery in our study. This conclusion is also supported by recent publications suggesting that the use of NMDAR antagonists might potentially prevent delirium (Bornemann‐Cimenti et al., 2016; Meco et al., 2023; Plyler et al., 2019; Roy et al., 2016). However, further research is needed to determine the specific NMDAR antagonist type, the optimal timing and dosage, and the duration of treatment that is most effective in preventing delirium. These aspects will require extensive investigation in future basic and clinical trials.

Recently, electroencephalograph studies of the brain's electrical activities of delirious patients suggested that neural network malfunction was involved in the occurrence of delirium (Kimchi et al., 2019; Shinozaki et al., 2018). In addition, it has been found that the synchronization between the pyramidal neurons and PV interneurons is tremendously important for the generation of theta and gamma oscillations, which play a critical role in the processes of perception, attention, and memory (Nunez & Buno, 2021). Given the function of the pyramidal neurons and PV interneurons were impaired in the aged mice after cardiac surgery, the alteration of theta and gamma oscillations found in our study may likely be the secondary pathogenesis of delirium‐like behavior in the aged mice after surgery. Further studies are needed to explore how the neuronal activities of the pyramidal and PV interneurons regulate brain oscillations and modulate behavioral changes after surgery. Once this is achieved, the promising perspectives of the earlier diagnosis and prompt treatment of POD will become possible.

In conclusion, a major traumatic surgery model was established in the present study to mimic clinical settings including cardiac surgery which enables us to study the pathogenesis of POD after surgery. The abnormal activities of the hippocampal neural networks induced by increased A1 astrocyte activation via enhancing NMDAR tonic activation in both the pyramidal neurons and PV interneurons were likely the key mechanisms mediating delirium‐like behavior in the aged mice following cardiac surgery. Our findings from molecular, cellular, and neuronal networks to behavioral changes reported here likely enhance our understanding of the underlying mechanisms of POD for further development of its prevention and therapeutic strategies. It is noteworthy that our study solely focused on the hippocampal changes in the “disease” model reported here. Whether the same mechanisms cause cortical dysfunction, which is also believed to contribute to delirium pathogenesis, is unknown and warrants further study.

## AUTHOR CONTRIBUTIONS

WXL conducted myocardial IR surgery; WXL and MJ carried out behavioral tests, IF, Golgi staining, and electrophysiological recording in vivo; WXL and JC did the electrophysiological recording in acute brain slices; KYZ, XYZ and RSL did the WB, RT‐qPCR and ELISA; YYZ did Microdialysis and HPLC, bred and took care of the animals in the study; ZJX, YPW and JP analyzed the data. WXL and MJ drafted the original manuscript. DQM, JJY, and DJW designed the study. All authors contributed to the article and approved the submitted version.

## FUNDING INFORMATION

This work was supported by grants from the National Natural Science Foundation of China (Nos. 81970401, 81971020, 82301368).

## CONFLICT OF INTEREST STATEMENT

The authors declared there were no potential competing interests in this study.

## Supporting information


Figure S1.

Figure S2.

Figure S3.

Figure S4.

Table S1.

Table S2.

Video S1.

Video S2.

Appendix S1.

## Data Availability

All raw data used in this manuscript are available on request.
