# Supplementary figures and images for: Increased A1 astrocyte activation‐driven hippocampal neural network abnormality mediates delirium‐like behavior in aged mice undergoing cardiac surgery
Source: Aging Cell. 2023 Dec 28;23(3):e14074. doi: 10.1111/acel.14074 (PMC10928578; doi:10.1111/acel.14074)

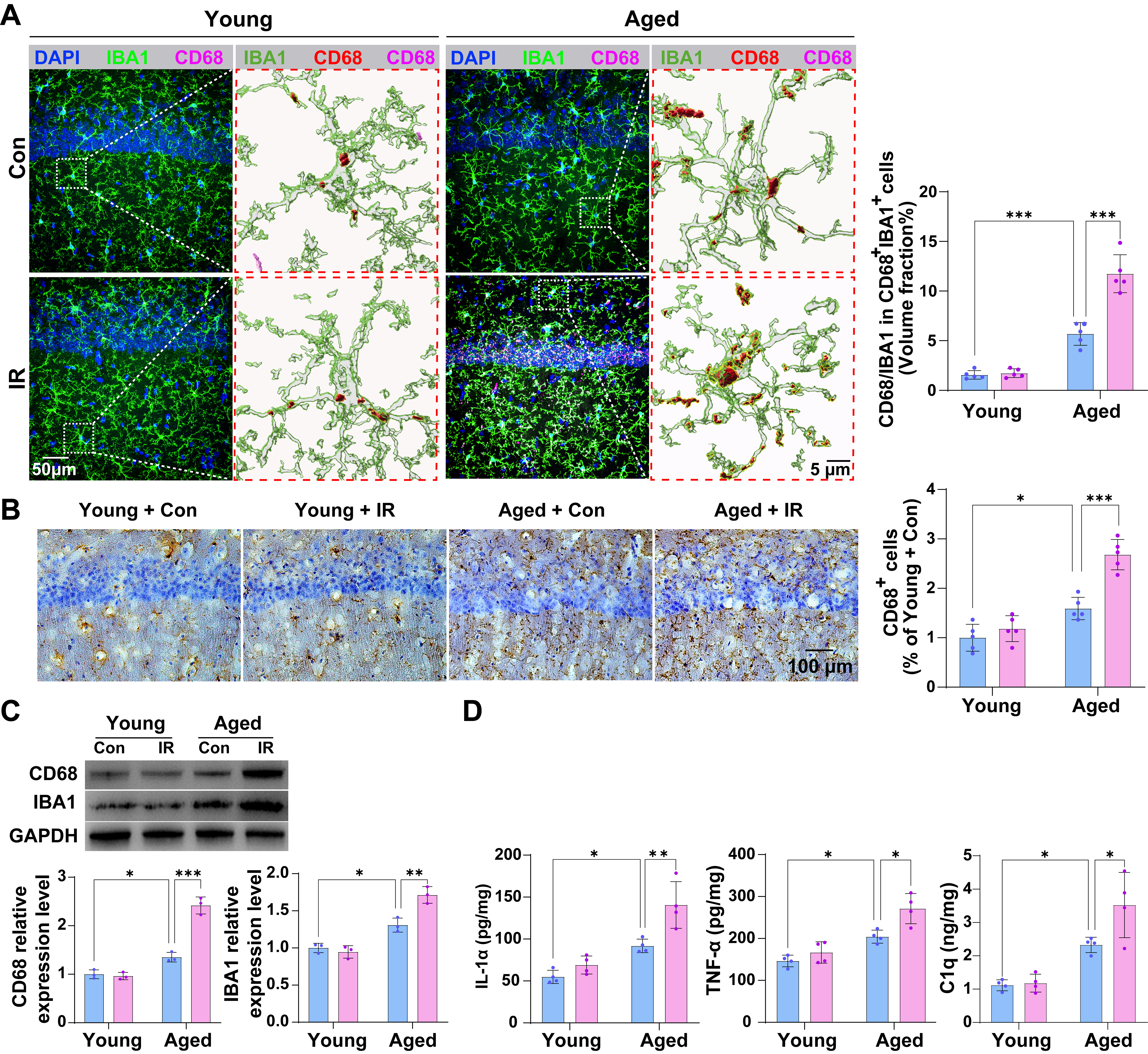

Supplement: Supplementary file 1 — Figure S1. [file ACEL-23-e14074-s005.tif]

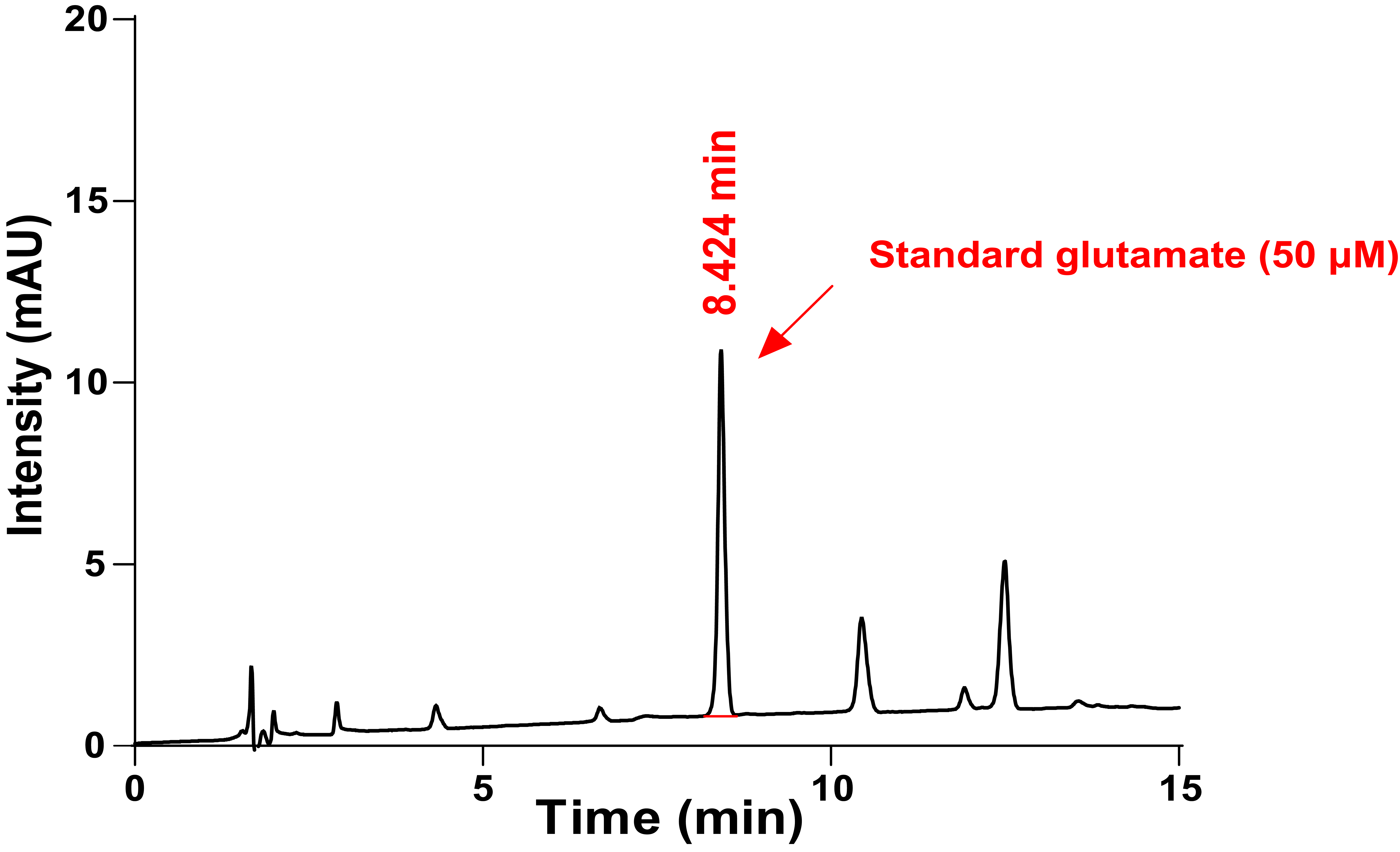

Supplement: Supplementary file 2 — Figure S2. [file ACEL-23-e14074-s006.tif]

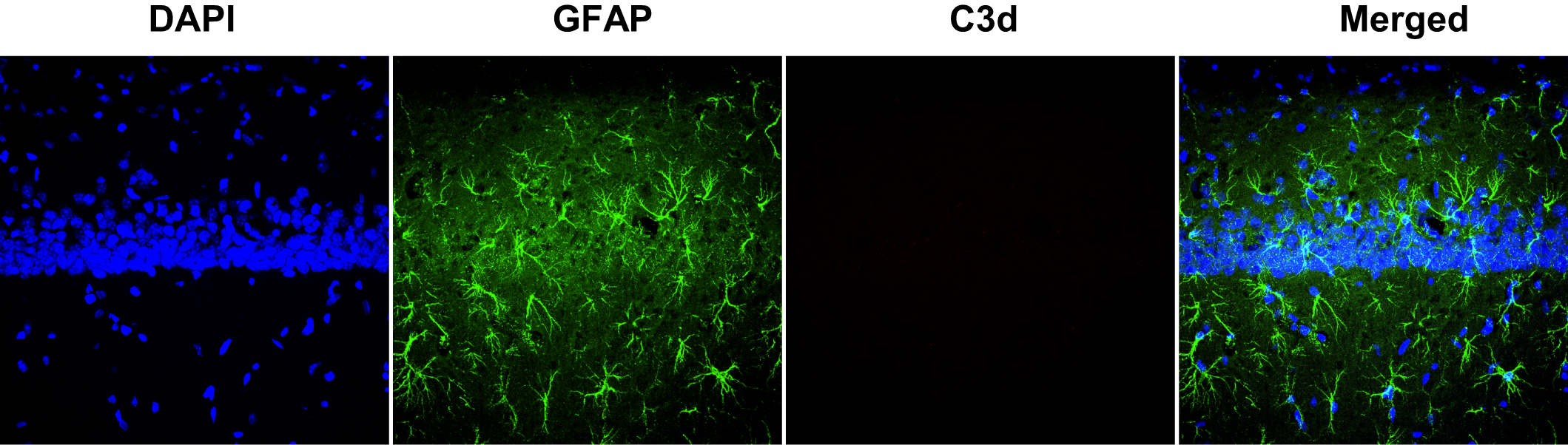

Supplement: Supplementary file 3 — Figure S3. [file ACEL-23-e14074-s009.tif]

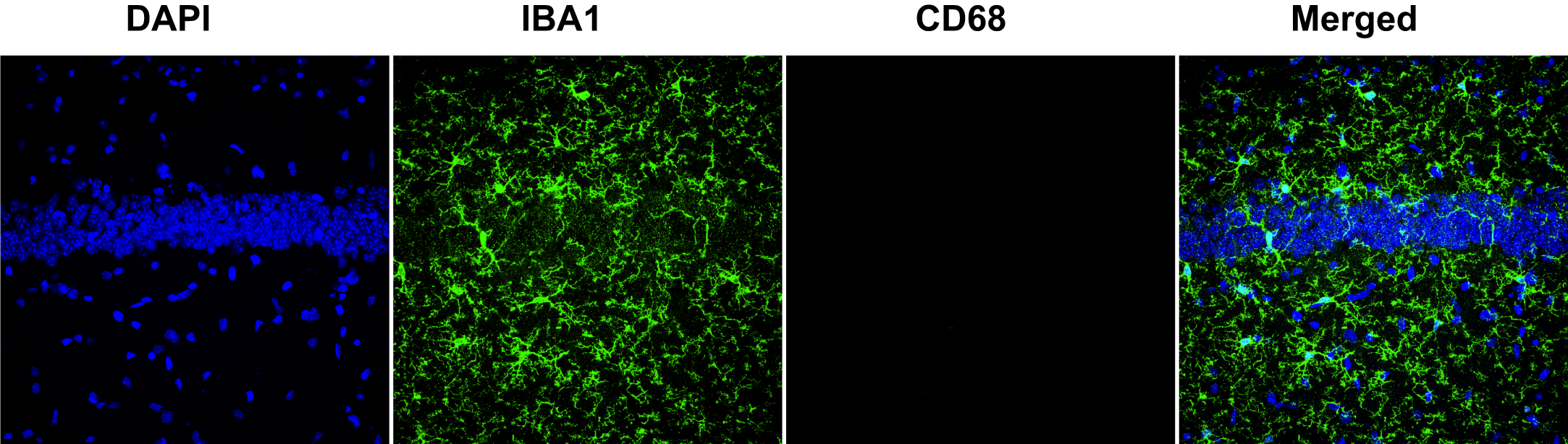

Supplement: Supplementary file 4 — Figure S4. [file ACEL-23-e14074-s002.tif]
