# Supplementary material for: Increased A1 astrocyte activation‐driven hippocampal neural network abnormality mediates delirium‐like behavior in aged mice undergoing cardiac surgery
Source: Aging Cell. 2023 Dec 28;23(3):e14074. doi: 10.1111/acel.14074 (PMC10928578; doi:10.1111/acel.14074)
Supplement: Supplementary file 5 — Table S1. [file ACEL-23-e14074-s004.docx]

**Table S1. qPCR primers for A1-special transcripts.**

| Primer | Forward sequence | Reverse sequence |
| --- | --- | --- |
| C3 | AAAAGGGGCGCAACAAGTTC | GATGCCTTCCGGGTTCTCAA |
| H2-T23 | GGACCGCGAATGACATAGC | GCACCTCAGGGTGACTTCAT |
| Serping1 | ACAGCCCCCTCTGAATTCTT | GGATGCTCTCCAAGTTGCTC |
| H2-D1 | TCCGAGATTGTAAAGCGTGAAGA | ACAGGGCAGTGCAGGGATAG |
| Ggta1 | GTGAACAGCATGAGGGGTTT | GTTTTGTTGCCTCTGGGTGT |
| Ligp1 | GGGGCAATAGCTCATTGGTA | ACCTCGAAGACATCCCCTTT |
| Gbp2 | GGGGTCACTGTCTGACCACT | GGGAAACCTGGGATGAGATT |
| Fbln5 | CTTCAGATGCAAGCAACAA | CCTATGGGTCACTTGCCACT |
| Ugt1a | CCTATGGGTCACTTGCCACT | AAAACCATGTTGGGCATGAT |
| Fkbp5 | TATGCTTATGGCTCGGCTGG | CAGCCTTCCAGGTGGACTTT |
| Psmb8 | CAGTCCTGAAGAGGCCTACG | CACTTTCACCCAACCGTCTT |
| Srgn | GCAAGGTTATCCTGCTCGGA | TGGGAGGGCCGATGTTATTG |
| Amigo2 | GAGGCGACCATAATGTCGTT | GCATCCAACAGTCCGATTCT |
