# Supplementary material for: Increased A1 astrocyte activation‐driven hippocampal neural network abnormality mediates delirium‐like behavior in aged mice undergoing cardiac surgery
Source: Aging Cell. 2023 Dec 28;23(3):e14074. doi: 10.1111/acel.14074 (PMC10928578; doi:10.1111/acel.14074)
Supplement: Supplementary file 6 — Table S2. [file ACEL-23-e14074-s001.docx]

| **Figure** | **Interaction** | | **Row Factor** | | **Column Factor** | |
| --- | --- | --- | --- | --- | --- | --- |
|  | **F (DFn, DFd)** | **P** | **F (DFn, DFd)** | **P** | **F (DFn, DFd)** | **P** |
| **Fig. 1A** | (1, 44) = 0.02448 | 0.8764 | (1, 44) = 30.47 | < 0.0001 | (1, 44) = 1.119 | 0.2959 |
| **Fig. 1B** | (1, 44) = 1.153 | 0.2887 | (1, 44) = 54.62 | < 0.0001 | (1, 44) = 26.11 | < 0.0001 |
| **Fig. 1C** | (1, 44) = 0.01183 | 0.9139 | (1, 44) = 0.7292 | 0.3978 | (1, 44) = 0.05696 | 0.8125 |
| **Fig. 1D** | (1, 44) = 2.86 | 0.0979 | (1, 44) = 9.423 | 0.0037 | (1, 44) = 7.184 | 0.0103 |
| **Fig. 1E** | (1, 44) = 0.3919 | 0.5345 | (1, 44) = 0.3292 | 0.5691 | (1, 44) = 0.3993 | 0.5307 |
| **Fig. 1F** | (1, 44) = 3.683 | 0.0615 | (1, 44) = 6.971 | 0.0114 | (1, 44) = 5.008 | 0.0303 |
| **Fig. 1G** | (1, 44) =  0.0000134 | 0.9971 | (1, 44) = 0.01744 | 0.8956 | (1, 44) = 0.4844 | 0.4901 |
| **Fig. 1H** | (1, 44) = 3.386 | 0.0725 | (1, 44) = 4.953 | 0.0312 | (1, 44) = 5.487 | 0.0237 |
| **Fig. 2A** | (1, 16) = 113.5 | < 0.0001 | (1, 16) = 532.7 | < 0.0001 | (1, 16) = 110.4 | < 0.0001 |
| **Fig. 2B** | (1, 16) = 37.76 | < 0.0001 | (1, 16) = 45.15 | < 0.0001 | (1, 16) = 69.01 | < 0.0001 |
| **Fig. 2C** | (1, 36) = 0.0002355 | 0.9878 | (1, 36) = 0.5014 | 0.4835 | (1, 36) = 25.27 | < 0.0001 |
| **Fig. 2D** | (1, 36) = 2.809 | 0.1024 | (1, 36) = 5.266 | 0.0277 | (1, 36) = 9.040 | 0.0048 |
| **Fig. 2E** | (1, 36) = 3.249 | 0.0798 | (1, 36) = 5.345 | 0.0266 | (1, 36) = 15.29 | 0.0004 |
| **Fig. 2F** | (1, 36) = 4.943 | 0.0326 | (1, 36) = 3.735 | 0.0612 | (1, 36) = 6.684 | 0.0139 |
| **Fig. 3A** | (1, 16) = 87.55 | <0.0001 | (1, 16) = 86.95 | <0.0001 | (1, 16) = 475.7 | <0.0001 |
| **Fig. 3B** | (1, 36) = 0.2176 | 0.6437 | (1, 36) = 0.5103 | 0.4796 | (1, 36) = 2.691 | 0.1096 |
| **Fig. 3C** | (1, 36) = 4.928 | 0.0328 | (1, 36) = 3.723 | 0.0616 | (1, 36) = 11 | 0.0021 |
| **Fig. 3D** | (1, 36) = 3.115 | 0.0861 | (1, 36) = 5.779 | 0.0215 | (1, 36) = 14.15 | 0.0006 |
| **Fig. 3E** | (1, 36) = 3.262 | 0.0793 | (1, 36) = 4.776 | 0.0355 | (1, 36) = 7.462 | 0.0097 |
| **Fig. 4A** | (1, 8) = 11.86 | 0.0088 | (1, 8) = 27.7 | 0.0008 | (1, 8) = 4.982 | 0.0561 |
| **Fig. 4B** | (1, 8) = 4.705 | 0.0619 | (1, 8) = 14.7 | 0.005 | (1, 8) = 8.015 | 0.0221 |
| **Fig. 4C-1** | (1, 92) = 0.03811 | 0.8456 | (1, 92) = 4.18 | 0.0438 | (1, 92) = 0.5407 | 0.4640 |
| **Fig. 4C-2** | (1, 68) = 3.119 | 0.0819 | (1, 68) = 15.87 | 0.0002 | (1, 68) = 5.102 | 0.0271 |
| **Fig. 4E-1** | (1, 8) = 5.961 | 0.0405 | (1, 8) = 9.149 | 0.0164 | (1, 8) = 6.323 | 0.0361 |
| **Fig. 4E-2** | (1, 8) = 16.51 | 0.0036 | (1, 8) = 14.73 | 0.005 | (1, 8) = 19.38 | 0.0023 |
| **Fig. 4E-3** | (1, 8) = 10.85 | 0.0109 | (1, 8) = 17.62 | 0.0030 | (1, 8) = 19.96 | 0.0021 |
| **Fig. 4F-1** | (1, 8) = 6.886 | 0.0305 | (1, 8) = 19.13 | 0.0024 | (1, 8) = 6.591 | 0.0333 |
| **Fig. 4F-2** | (1, 8) = 2.586 | 0.1465 | (1, 8) = 32.71 | 0.0004 | (1, 8) = 16.72 | 0.0035 |
| **Fig. 5A** | (1, 23) = 7.173 | 0.0134 | (1, 23) = 7.948 | 0.0097 | (1, 23) = 42.64 | <0.0001 |
| **Fig. 5B-1** | (1, 22) = 5.872 | 0.0241 | (1, 22) = 2.955 | 0.0996 | (1, 22) = 9.772 | 0.0049 |
| **Fig. 5B-2** | (1, 22) = 0.0159 | 0.9008 | (1, 22) = 0.01597 | 0.9006 | (1, 22) = 0.3469 | 0.5619 |
| **Fig. 5D** | (1, 23) = 6.037 | 0.022 | (1, 23) = 6.091 | 0.0215 | (1, 23) = 44.99 | <0.0001 |
| **Fig. 5E-1** | (1, 26) = 6.118 | 0.0202 | (1, 26) = 3.878 | 0.0597 | (1, 26) = 14.42 | 0.0008 |
| **Fig. 5E-2** | (1, 26) = 0.000003663 | 0.9985 | (1, 26) = 1.378 | 0.2511 | (1, 26) = 0.2972 | 0.5903 |
| **Fig. 6C-1** | (1, 12) = 3.988 | 0.069 | (1, 12) = 6.779 | 0.0231 | (1, 12) = 7.842 | 0.016 |
| **Fig. 6C-2** | (1, 12) = 4.985 | 0.0454 | (1, 12) = 12.16 | 0.0045 | (1, 12) = 9.864 | 0.0085 |
| **Sup. 1A** | (1, 16) = 32.44 | <0.0001 | (1, 16) = 187.2 | <0.0001 | (1, 16) = 36.32 | <0.0001 |
| **Sup. 1B** | (1, 16) = 14.35 | 0.0016 | (1, 16) = 75.91 | <0.0001 | (1, 16) = 28.13 | <0.0001 |
| **Sup. 1C-1** | (1, 8) = 66.92 | <0.0001 | (1, 8) = 179.7 | <0.0001 | (1, 8) = 58.30 | <0.0001 |
| **Sup. 1C-2** | (1, 8) = 19.36 | 0.0023 | (1, 8) = 106.1 | <0.0001 | (1, 8) = 11.40 | 0.0097 |
| **Sup. 1D-1** | (1, 12) = 4.699 | 0.0510 | (1, 12) = 46.21 | <0.0001 | (1, 12) = 15.56 | 0.0019 |
| **Sup. 1D-2** | (1, 12) = 3.683 | 0.0791 | (1, 12) = 44.64 | <0.0001 | (1, 12) = 12.81 | 0.0038 |
| **Sup. 1D-3** | (1, 12) = 4.628 | 0.0525 | (1, 12) = 45.41 | <0.0001 | (1, 12) = 5.653 | 0.0349 |

Note: Fig. 4C-1, Total dendritic length; Fig 4C-2, Number of branching points; Fig. 4E-1, PSD95 relative expression level; Fig. 4E-2, GluA1 relative expression level; Fig. 4E-3, GluA2 relative expression level; Fig. 4F-1, Relative PV fluorescence intensity; Fig. 4F-2, Relative GAD65/67 fluorescence intensity; Fig. 5B-1, mEPSCs inter-event interval; Fig. 5B-2, mEPSCs amplitude; Fig. 5E-1, mIPSCs inter-event interval; Fig. 5E-2, mIPSCs amplitude. Fig. 6C-1, Theta power; Fig. 6C-2, Gamma power; Sup. 1C-1, CD68 relative expression level; Sup. 1C-2, IBA1 relative expression level; Sup. 1D-1, IL-1α; Sup. 1D-2, TNF-α; Sup. 1D-3, C1q.
