# Supplementary material for: Increased A1 astrocyte activation‐driven hippocampal neural network abnormality mediates delirium‐like behavior in aged mice undergoing cardiac surgery
Source: Aging Cell. 2023 Dec 28;23(3):e14074. doi: 10.1111/acel.14074 (PMC10928578; doi:10.1111/acel.14074)
Supplement: Supplementary file 9 — Appendix S1. [file ACEL-23-e14074-s007.docx]

**Figure S1.** Cardiac surgery leads to significant activation of microglia in the hippocampus of aged mice. **A** Representative confocal images of IBA1 (Green), C68 (Magenta) and DAPI (Blue) and 3D rendering of IBA1 (Cyan), CD68 inside GFAP (Red) and CD68 outside GFAP (Magenta). The activation level of microglia was evaluated by measuring the volume fraction of CD68 inside IBA1 to IBA1. **B** The number of CD68 positive cells was determined by IHC. **C** The expression levels of CD68 and IBA1 were determined by WB. **D** The levels of IL-1α, TNF-α and C1q in the hippocampus were detected by ELISA. Data are expressed as mean ± SD (n = 5 per group for IF and IHC, n=3 for WB, n = 4 per group for ELISA). Two-way ANOVA, followed by Bonferroni post hoc for multiple comparisons, was used to analyze the data. ^*^*P* < 0.05, ^**^*P* < 0.01 and ^***^*P* < 0.001.

**Figure S2.** The retention time of glutamate in the chromatographic column was assessed by 50 μM standard glutamate.

**Figure S3.** Negative control for secondary antibody to C3d.

**Figure S4.** Negative control for secondary antibody to CD68.

**Video S1.** Representative Z-stack and 3D reconstruction video for GFAP, C3d and DAPI. The cell is from the Aged + IR group in Figure 2A.

**Video S2.** Representative Z-stack and 3D reconstruction video for IBA1, CD68 and DAPI. The cell is from the Aged +IR group in Supplementary Fig. 1A.
